# Trends and Disparities in the Incidence of Intraocular Foreign Bodies 1990–2019: A Global Analysis

**DOI:** 10.3389/fpubh.2022.858455

**Published:** 2022-06-20

**Authors:** Minjie Yuan, Qianyi Lu

**Affiliations:** ^1^Department of Ophthalmology, Eye Center of the Second Affiliated Hospital, School of Medicine, Zhejiang University, Hangzhou, China; ^2^Department of Ophthalmology, The First Affiliated Hospital of Soochow University, Suzhou, China

**Keywords:** intraocular foreign bodies, trends, epidemiology, Global Burden of Disease Study (GBD), incidence

## Abstract

**Objectives:**

This study aims to provide trends and disparities in the incidence of intraocular foreign bodies (IOFBs) from 1990 to 2019 in 204 countries by region, country, socio-demographic index (SDI), age, and sex.

**Methods:**

The global, regional and national number of incident cases as well as age-standardized incidence rate (ASIR) of IOFBs were attained from the Global Burden of Disease Study 2019 (GBD 2019). To estimate the trend of ASIR of IOFBs, the estimated annual percentage change (EAPC) was calculated from 1990 to 2019.

**Results:**

Globally, although ASIR of IOFBs decreased with an EAPC of −0.93% [95% uncertainty interval (UI) −1.1 to −0.76] from 1990 to 2019, ASIR of IOFBs increased from 2008 to 2019. From 35.79 million (95% UI 23.62–50.89) in 1990 to 46.63 million (95% UI 32.45–64.45) in 2019, the number of IOFB incident cases worldwide increased by 30.29% (95% UI 19.63–43.55). The incidence of IOFBs varied by region and country, and it was closely related to socio-economic development. Furthermore, while ASIR of IOFBs was high in the young population aged 15–49 years, we observed a significant increase in the number of IOFB incident cases in older adults when compared to other age groups. In terms of sex, males accounted for the vast majority of IOFB incident cases.

**Conclusions:**

The global ASIR of IOFBs is on the rise, with an increase in incident cases, designating IOFBs as a global health challenge. The incidence of IOFBs cases is directly related to geographic location, socio-economic status, age, sex, and other factors. Our findings could be useful for the control and prevention of IOFBs.

## Introduction

Open Globe Injury (OGI) is a leading cause of unilateral visual impairment in adolescents and young adults (1). It imposed an immense burden on society and individuals. Recent data indicate the economic limitation of $793 million associated with OGI in the United States (2). In some African countries, ocular trauma places a tremendous burden on individuals due to the realities of low density of health facilities, few ophthalmologists, and little access to eye care (3–5). Intraocular foreign bodies (IOFBs), which account for 18% to 41% of all OGI, are unintentional projectiles retained in the eye that require urgent diagnosis and treatment to prevent blindness or loss of the globe (6–8). IOFBs occurred primarily in men and workplaces, with the most common mechanism of injury being metal-to-metal work, farm work, fireworks, explosions, and gunshots (9–11).

Over the last few decades, the incidence and tendency of IOFBs may have been influenced by structural changes in the economy, improvements in living circumstances, changes in the work environment, increase in socio-demographic status and life expectancy (12). Previous reports, however, have limitations, due to small study populations (11), analyzing only emergency department visits (13), or using other indicators for analysis (14).

To inform public health policy and prevention strategies for eye injuries, a comprehensive study of the global, regional, and national incidence of IOFBs, as well as their changing trends, is required. Based on the Global Burden of Disease Study 2019 (GBD 2019), the variations in the global incidence of IOFBs by region, country, Socio-demographic Index (SDI), age, and sex between 1990 and 2019 was investigated in this study. As an imperative complement to previous studies, our findings will provide valuable insights for evidence-based health care planning and resource allocation for IOFBs prevention to reduce the global incidence of IOFBs.

## Methods

### Overview

GBD 2019 estimated the incidence, prevalence, mortality, years of life lost (YLLs), years lived with disability (YLDs), and disability-adjusted life-years (DALYs) due to 369 diseases and injuries, in 204 countries from 1990 to 2019. GBD 2019 methods and results have been extensively documented in GBD literature (15). In summary, the primary sources of data for IOFBs were epidemiologic surveillance systems, registries, and published literature. Disease Modeling-Meta Regression (DisMod-MR) version 2.1, a Bayesian meta-regression framework widely used for GBD epidemiological modeling, was used to model the epidemiological outcomes of IOFBs. This framework integrates prevalence, incidence, remission, and mortality data into a single model.

### Data Source

The following IOFB data were obtained from the most recent version of the GBD study using the Global Health Data Exchange (GHDx) query tool (http://ghdx.healthdata.org/gbd-results-tool): (1) global, GBD region-, SDI region- and country-specific incidence data from 1990 to 2019 as absolute number and age-standardized rate (ASR); (2) age-specific incidence data from 1990 to 2019, with ASR calculated in absolute numbers for different age groups (0–14, 15–49, 50–69, 70+); (3) sex-specific incidence data from 1990 to 2019, as absolute number and ASR. This study did not require ethics approval or informed consent because the data was freely available to the public.

### Geographical Stratifications and Socio-Demographic Index

GBD divides countries and territories geographically into seven super regions (South-East Asia, East Asia and Oceania; sub-Saharan Africa (SSA); South Asia; Latin America and Caribbean; North Africa and Middle East; Central Europe, Eastern Europe and Central Asia; and High-income). A more detailed geographic stratification is shown in Supplementary Figure S1.

The SDI is a summary index that is used to determine where a country or geographical area falls on the development spectrum (15). The SDI is a composite average ranking of income per capita, average educational attainment, and fertility rates for all regions in the GBD study expressed on a scale of 0 to 1. SDI from the GHDx categorizes countries and territories into five categories (high, high-middle, middle, low-middle, and low SDI). Supplementary Figure S2 shows the detailed SDI groupings by country.

### Study Variables

Number of incident cases, change in number of incident cases, age-standardized incidence rate (ASIR) and the estimated annual percentage change (EAPC) by sex, region, SDI region and country from 1990 to 2019, and 95% uncertainty intervals (UI) for presentation of study results.

### Statistical Analysis

The GBD database was used to extract annual data on the occurrence of IOFBs. When considering differences in the age structure, the ASR is a crucial and representative indicator based on the age structure of a standard population of multiple populations (15). The ASR was calculated using the following formula:


$$\begin{array}{l} ASR\;\left(per\;100,000\;population\right)\;=\;\frac{\overset{A}{\underset{i=1}{\sum }}a_{i}w_{i}}{\overset{A}{\underset{i\;=\;1}{\sum }}w_{i}}\times 100,000 \end{array}$$

In the formula, *a*_*i*_ is the rate in the *i*^th^ age group and *w*_*i*_ is a number of GBD standard populations in the corresponding *i*^th^ age subgroup. Furthermore, the EAPC is a summary measure commonly used for ASR trends within a given interval (16). The regression line evaluated the natural logarithm of the rate, e.g., *y* = α+β*x*+ε, where *y* = ln(*ASR*), and x is the calendar year. The EAPC was calculated as 100 × [exp(β)−1], along with a 95% UI with a linear regression model. All analyses and data visualizations were accomplished using the R program (version 4.0.0).

## Results

### Global Level

**Figure 1:**
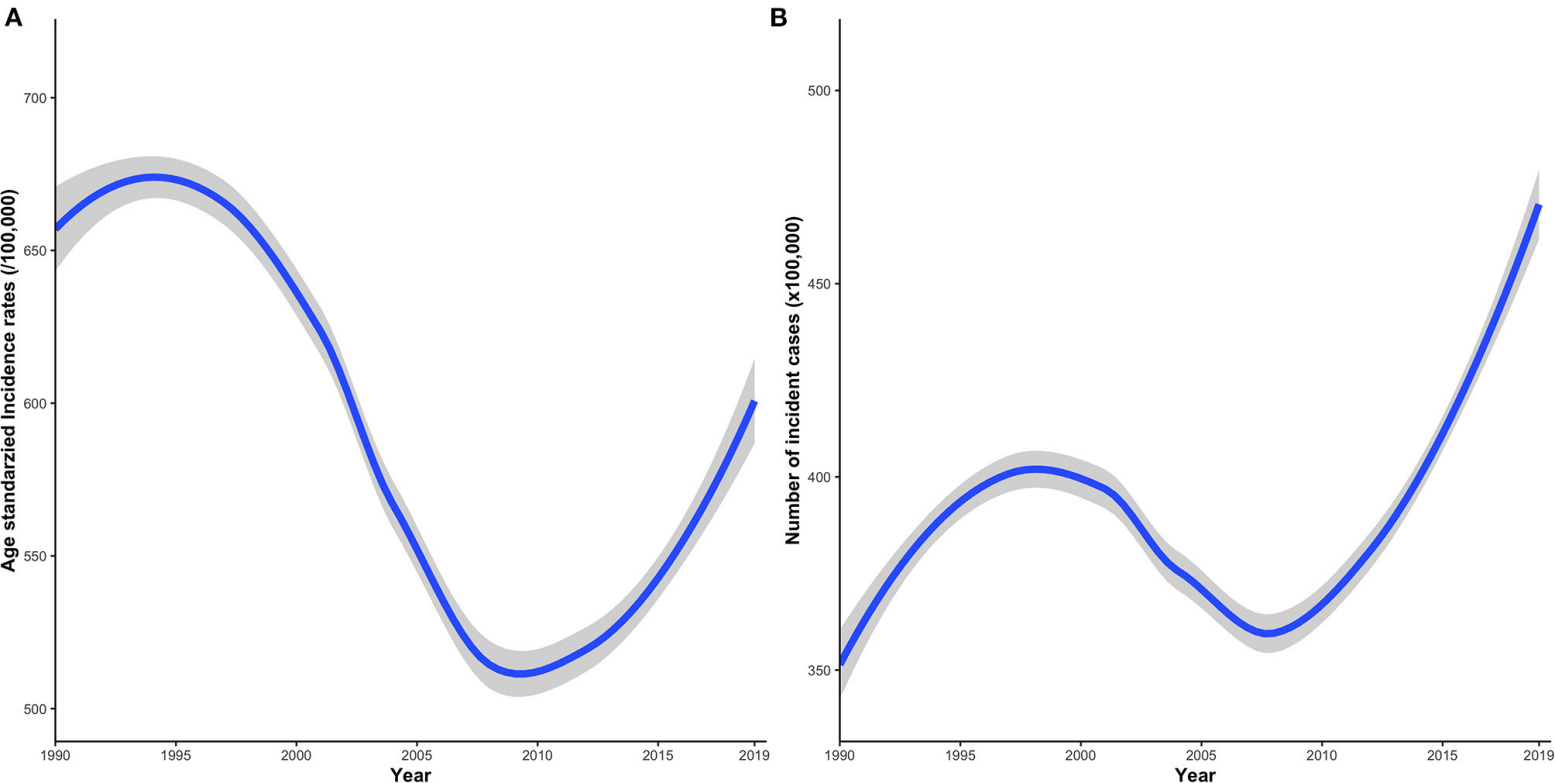
Time trends of the global disease incidence of IOFBs from 1990 to 2019. **(A)** IOFBs burden in terms of ASIR per 100,000 persons; **(B)** IOFBs burden in terms of the number of incident cases × 100,000 persons. IOFBs, intraocular foreign bodies; ASIR, age-standardized incidence rate.

This study revealed that globally ASIR of IOFBs decreased with an EAPC of −0.93% (95% UI −1.1 to −0.76) from 665.92 (95% UI 442.23–944.1) per 100 000 population in 1990 to 593.26 (95% UI 416.01–812.51) per 100 000 population in 2019 (Table 1). However, the ASIR of IOFBs showed a growing trend from 2008 to 2019 (Figure 1A). The number of incident cases of IOFBs increased by 30.29% (95% UI 19.63–43.55), from 35.79 million (95% UI 23.62 to 50.89) in 1990 to 46.63 million (95% UI 32.45–64.45) in 2019 (Table 1). Notably, the number of IOFBs incident cases increased significantly after 2008 (Figure 1B).

**Table 1:** Estimated number and age-standardized rate (per 100,000 persons) of incidence and corresponding trend for IOFBs from 1990 to 2019 by sex, age group, SDI, and GBD regions.

**Characteristics**	**Number (95% UI)**		**1990–2019 number of change (%) (95% UI)**	**ASIR (95% UI)**		**1990–2019 EAPC (95% UI)**
	**1990**	**2019**		**1990**	**2019**	
**Global**	35,789,179.47 (23,616,153.33, 50,893,362.63)	46,628,656.11 (32,450,161.35, 64,454,053.45)	30.29 (19.63, 43.55)	665.92 (442.23, 944.1)	593.26 (416.01, 812.51)	−0.93 (−1.1, −0.76)
**Sex**						
Male	25,685,025.95 (16,747,388.19, 36,937,800.73)	32,415,392.02 (22,199,368.36, 45,160,008.79)	26.2 (15.45, 40.34)	942.83 (619.16, 1348.31)	817.28 (564.55, 1128.95)	−1.07 (−1.21, −0.92)
Female	10,104,153.51 (6,900,359.98, 14,180,961.08)	14,213,264.08 (10,056,222.85, 19,488,644.18)	40.67 (30.23, 51.59)	382.56 (262.4, 536.84)	365.09 (260.26, 500.61)	−0.58 (−0.8, −0.37)
**Age group**						
0–14 years	7,296,298.12 (3,463,712.37, 13,814,678.03)	7,532,632.1 (3,674,158.38, 13,910,515.55)	3.24 (−1.76, 9.78)	418.34 (198.16, 793.23)	382.09 (186.81, 704.61)	−0.61 (−0.82, −0.4)
15–49 years	24,500,477.09 (9,296,584.07, 49,838,371.45)	31,457,002.45 (12,999,221.69, 61,501,304.18)	28.39 (16.1, 44.08)	909.92 (344.23, 1853.24)	796.81 (329.77, 1555.79)	−1.03 (−1.17, −0.88)
50–69 years	3,536,888.01 (1,440,813.14, 7,258,397.42)	6,640,587.64 (2,732,603.99, 13,591,997.45)	87.75 (84.32, 91.43)	517.35 (210.66, 1061.92)	479.92 (197.67, 982.01)	−0.93 (−1.13, −0.74)
70+ years	455,516.24 (218,193.23, 879,971.69)	998,433.92 (482,670.67, 1,917,854.38)	119.19 (112.1, 128.46)	218.07 (104.52, 421.47)	212.15 (102.62, 407.38)	−0.64 (−0.93, −0.35)
**SDI region**						
High SDI	4,046,569.65 (2,877,669.07, 5,545,126.9)	4,595,376.8 (3,278,208.41, 6,319,374.82)	13.56 (8.01, 20.92)	489.4 (349.18, 668.64)	480.6 (343.03, 654.35)	−0.3 (−0.5, −0.11)
High-middle SDI	8,008,800.3 (5,053,205.96, 1,1918,030.94)	8,710,343.99 (5,612,005.44, 12,983,283.86)	8.76 (−4.04, 24.53)	676.19 (432.07, 990.75)	585.94 (383.49, 842.16)	−1.24 (−1.41, −1.07)
Middle SDI	13,736,127.12 (8,318,310.04, 20,789,728.55)	15,525,470.47 (10,257,245.22, 22,458,313.78)	13.03 (−0.62, 32.74)	801.74 (490.81, 1207.44)	620.81 (414.59, 874.47)	−1.5 (−1.67, −1.34)
Low-middle SDI	7,168,944.32 (5,089,976.18, 9,750,252.58)	10,987,779.15 (7,971,830.49, 14,819,381.64)	53.27 (43.49, 61.51)	647.18 (468.36, 879.07)	605.82 (441.81, 815.39)	−0.4 (−0.57, −0.23)
Low SDI	2,816,276.53 (2,071,208.56, 3,820,851.3)	5,664,574.98 (4,125,839.02, 7,661,431.16)	101.14 (96.12, 105.2)	559.24 (415.73, 748.91)	511.39 (379.99, 686.92)	−0.08 (−0.26, 0.1)
**South–East Asia, East Asia and Oceania**						
East Asia	13,016,642.89 (6,581,803.13, 22,143,406.43)	11,380,587.45 (5,800,404.29, 19,207,306.61)	−12.57 (−28.65, 10.9)	1026.83 (538, 1704.6)	702.42 (371.02, 1164.97)	−3.4 (−3.56, −3.25)
South-East Asia	1,633,547.91 (1,187,805.87, 2,235,629.61)	2,520,734.71 (1,830,480.45, 3,461,866.92)	54.31 (41.8, 67.58)	349.5 (255.14, 473.89)	357.71 (262.09, 485.72)	0.09 (−0.13, 0.31)
Oceania	19,529.55 (14,059.12, 26,797.95)	41,060.21 (29,975.53, 56,789.06)	110.25 (102.69, 117.13)	310.16 (225.22, 425.93)	308.79 (224.42, 423.84)	−0.01 (−0.25, 0.23)
**Sub-Saharan Africa**						
Southern	283,833.25 (204,563.59, 390,099.95)	446,606.2 (324,523.2, 609,680.94)	57.35 (44.64, 67.87)	545.74 (404.53, 740.46)	547.66 (405.79, 743.38)	0.02 (−0.15, 0.2)
Western	927,283.3 (683,595.02, 1,268,597.31)	2,234,038.67 (1,626,929.08, 3,041,574.86)	140.92 (136.99, 144.04)	509.25 (376.01, 683.76)	499.46 (368.94, 671.04)	−0.07 (−0.26, 0.11)
Central	221,478.93 (161,746.66, 300,779.51)	542,831.99 (397,649.46, 742,420.2)	145.09 (139.98, 149.89)	417.02 (309.46, 562.86)	418.53 (310.69, 564.13)	0.01 (−0.19, 0.22)
Eastern	881,323.15 (639,497.68, 1,213,007.26)	1,986,279.43 (1,432,940.26, 2,712,605.21)	125.37 (118.94, 130.88)	488.15 (360.42, 656.74)	488.91 (361.08, 658.11)	−0.01 (−0.19, 0.18)
**South Asia**						
South Asia	7,789,753.64 (5,680,232.24, 10,430,149.35)	13,324,346.41 (9,695,190.99, 17,897,286.43)	71.05 (61.62, 78.83)	714.09 (529.01, 961.1)	711.12 (526.75, 957.21)	−0.02 (−0.17, 0.14)
**Latin America and Caribbean**						
Caribbean	182,696.78 (132,442.5, 245,813.68)	243,045.65 (178,024.61, 328,427.41)	33.03 (25.49, 41.31)	510.52 (375.45, 689.08)	511.74 (376.05, 690.44)	0.01 (−0.17, 0.19)
Central	1,024,695.08 (738,900.55, 1,378,378.13)	1,582,598.13 (1,162,642.62, 2,139,817.25)	54.45 (41.68, 66.65)	621.12 (458.13, 839.01)	617.34 (454.84, 834.69)	−0.02 (−0.19, 0.14)
Tropical	1,124,079.15 (817,016.94, 1,502,667.02)	1,659,804.72 (1,211,793.22, 2,236,266.35)	47.66 (34.68, 60.64)	720.35 (532.89, 969.68)	719.29 (532.06, 968.4)	0 (−0.16, 0.15)
Andean	195,107.5 (140,473.07, 264,719.81)	333,075.81 (242,696.86, 448,120.37)	70.71 (58.06, 82.08)	511.25 (375.96, 690.29)	513.1 (376.85, 691.66)	0.01 (−0.17, 0.2)
**North Africa and Middle East**						
North Africa and Middle East	1,885,683.65 (1,364,532.98, 2,558,785.13)	3,519,116.24 (2,559,918.05, 4,768,904.17)	86.62 (67.61, 103.5)	552.99 (405.78, 746.89)	550.67 (404.14, 746.79)	0 (−0.18, 0.17)
**Central Europe, Eastern Europe and Central Asia**						
Central Asia	332,836.01 (240,416.34, 454,800.37)	457,733.97 (336,079.91, 627,518.74)	37.53 (29.46, 46.02)	468.79 (345.29, 639.4)	471.45 (347.86, 642.08)	0.02 (−0.17, 0.21)
Eastern Europe	1,381,008.87 (1,008,839.29, 1,861,676.54)	1,228,442.74 (903,906.3, 1,663,544.31)	−11.05 (−15.58, −6.21)	614.67 (451.27, 837.59)	618.77 (454.69, 843.24)	0.02 (−0.15, 0.19)
Central Europe	586,637.92 (431,215.76, 790,619.5)	516,034.8 (376,752.52, 694,795.61)	−12.04 (−17.91, −6.35)	479.82 (352.54, 648.84)	486.64 (357.57, 659.68)	0.05 (−0.14, 0.24)
**High–income regions**						
Southern Latin America	157,587.19 (115,518.52, 213,085.36)	211,113.2 (155,218.09, 283,467.83)	33.97 (26.42, 39.36)	317.26 (232.37, 427.57)	317.56 (232.62, 428.22)	−0.01 (−0.25, 0.22)
Western Europe	1,364,171.42 (1,002,594.8, 1,837,022.34)	1,429,442.33 (1,054,555.58, 1,916,914.45)	4.78 (0.02, 10.78)	366.17 (271.08, 498.32)	367.1 (271.8, 499.36)	0.04 (−0.18, 0.26)
North America	1,673,772.7 (1,102,264.62, 2,476,802.83)	1,894,415.52 (1,260,086.03, 2,754,042.83)	13.18 (4.87, 22.19)	587.93 (387.59, 853.13)	545.96 (360.7, 790.68)	−1.01 (−1.21, −0.81)
Australasia	96,510.14 (70,583.33, 130,076.89)	128,357.86 (95,133.65, 172,119.66)	33 (28.23, 40.27)	469.56 (347.24, 634.75)	465.33 (344.09, 629.24)	−0.03 (−0.22, 0.16)
Asia Pacific	1,011,000.42 (742,083, 1,362,581.84)	948,990.06 (702,143.06, 1,264,394.62)	−6.13 (−12.24, 1.2)	570.62 (422.82, 770.02)	562.67 (416.19, 760.08)	−0.05 (−0.22, 0.12)

*IOFBs, intraocular foreign bodies; SDI, socio-demographic index; UI, uncertainty interval; ASIR, age-standardized incidence rate; EAPC, estimated annual percentage change*.

### Regional Level

**Figure 2:**
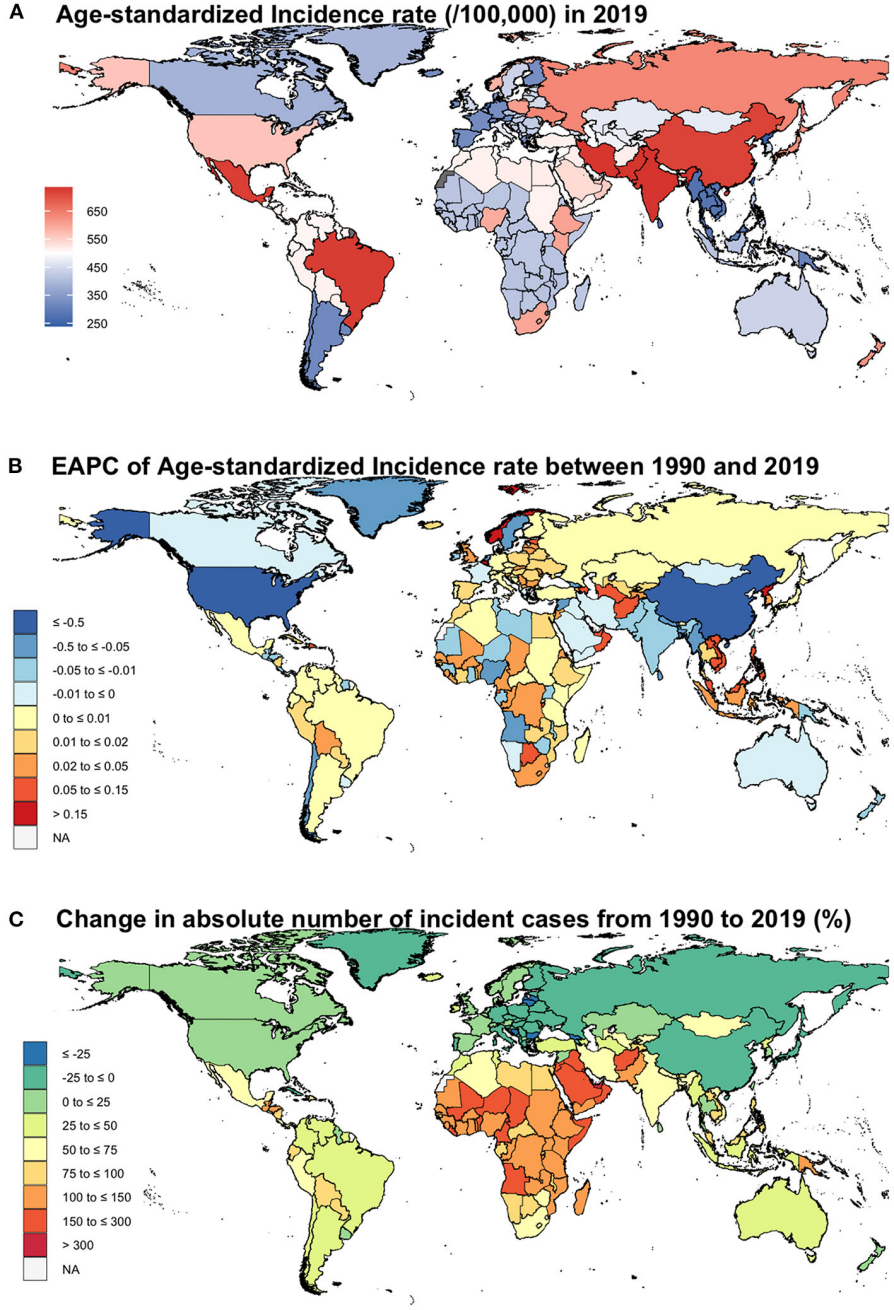
Choropleth maps showing geographic variation in ASIR of IOFBs. **(A)** ASIR per 100,000 persons in 2019; **(B)** EAPC of ASIR between 1990 and 2019; **(C)** Change in absolute number of incident cases from 1990 to 2019 (%). ASIR, age-standardized incidence rate; IOFBs, intraocular foreign bodies; EAPC, estimated annual percent change.

Tropical Latin America, South Asia, and East Asia had the highest ASIR of IOFBs in 2019 at the regional level (Figure 2A). On the other hand, Oceania, Southern Latin America, and Southeast Asia had the lowest ASIR of IOFBs in 2019. Between 1990 and 2019, the ASIR decreased primarily in East Asia and high-income North America with an EAPC of −3.4 (95% UI −3.56 to −3.25) and −1.01 (95% UI −1.21 to −0.81), respectively, whereas the rest of the regions displayed fluctuating trends (Table 1; Figure 2B). In 2019, the number of IOFBs incident cases was highest in South Asia, East Asia, and North Africa and Middle East, while lowest in Oceania, Australasia, and Southern Latin America (Table 1). Between 1990 and 2019, IOFBs incident cases in East Asia, Central Europe, and Eastern Europe decreased significantly (Figure 2C). However, the most significant increase in incident cases was observed from 1990 to 2019 in Central sub-Saharan Africa, Western sub-Saharan Africa, and Eastern sub-Saharan Africa.

### National Level

In 2019, the ASIR of IOFBs ranged from 241.41 to 735.29 per 100,000 population across 204 countries (Supplementary Table S1). India, Pakistan, and Iran showed the highest ASIRs for IOFBs in 2019, while the Democratic People's Republic of Korea, the Netherlands, and Myanmar had the lowest ASIRs for IOFBs. In addition, countries with the highest number of incident cases of IOFBs in 2019 were China, India, and the United States of America, in exact order, while the lowest number of incident cases of IOFBs were in the following countries Tokelau, Niue, and Nauru. From 1990 to 2019, the EAPC of IOFBs varied by country (Supplementary Table S1). Between 1990 and 2019, only three countries with decreasing trends in ASIR of IOFBs were China, the United States of America, and Sweden. Nonetheless, Belgium, Maldives, and Norway were the only countries to show positive trends during that period. The remaining countries exhibited a volatile trend.

### The Incidence of IOFBs by SDI

**Figure 3:**
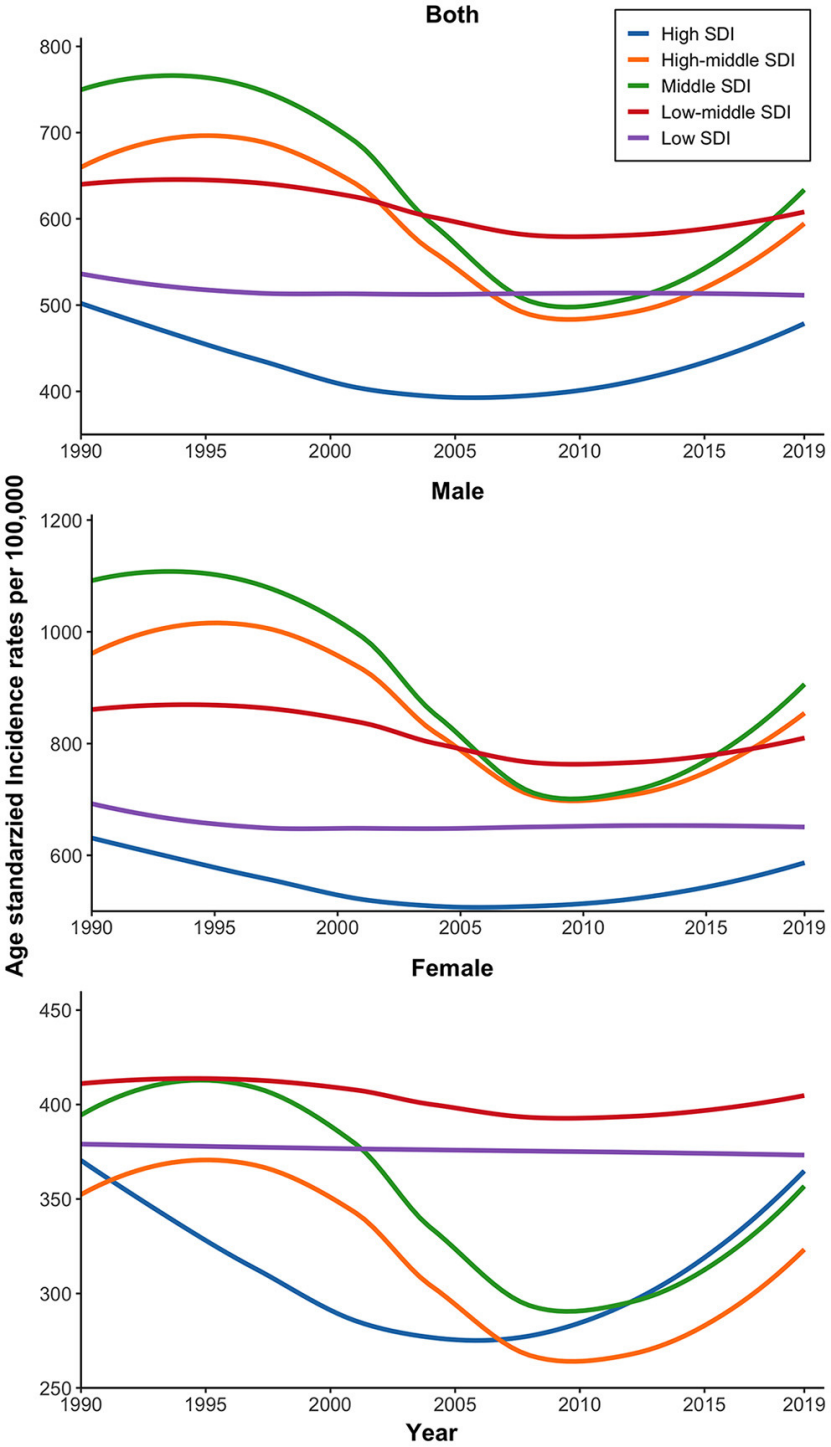
Time trends of the global burden of IOFBs in terms of ASIR (per 100,000 persons) from 1990 to 2019 by SDI and sex. IOFBs, intraocular foreign bodies; ASIR, age-standardized incidence rate; SDI, socio-demographic index.

As shown in Table 1, the middle SDI region had the highest ASIR of IOFBs in 2019, followed by the low-middle SDI region, high-middle SDI region, and low SDI region, and the high SDI region had the lowest (Supplementary Figure S2 shows the detailed SDI groupings by country). In 2019, the number of incident cases of IOFBs was highest in the middle SDI region and lowest in the high SDI region. Figure 3 represents an overview of ASIR for IOFBs by SDI from 1990 to 2019. Between 1990 and 2008, the ASIR of IOFBs in the middle, high-middle, and high SDI regions decreased but then increased steadily after 2008 until 2019. In contrast, the ASIR of IOFBs remained stable from 1990 to 2019 in the low and low-middle SDI regions. Notably, the low SDI region [101.14% (95% UI 96.12–105.2)] had a tremendous increase in the number of IOFBs changes from 1990 to 2019 (Table 1).

### Age and Sex Patterns

Globally, the highest ASIR and the number of incident cases of IOFBs in 2019 were reported in the age group 15–49, while the 70+ age group had the lowest ASIR and the number of incident cases (Table 1). From 1990 to 2019, the EAPC of IOFBs declined for all age groups, with the most pronounced decline in the age group 15–49 (Table 1). Notably, the number of changes in IOFBs increased with age between 1990 and 2019, and was most pronounced in the 70+ age group.

**Figure 4:**
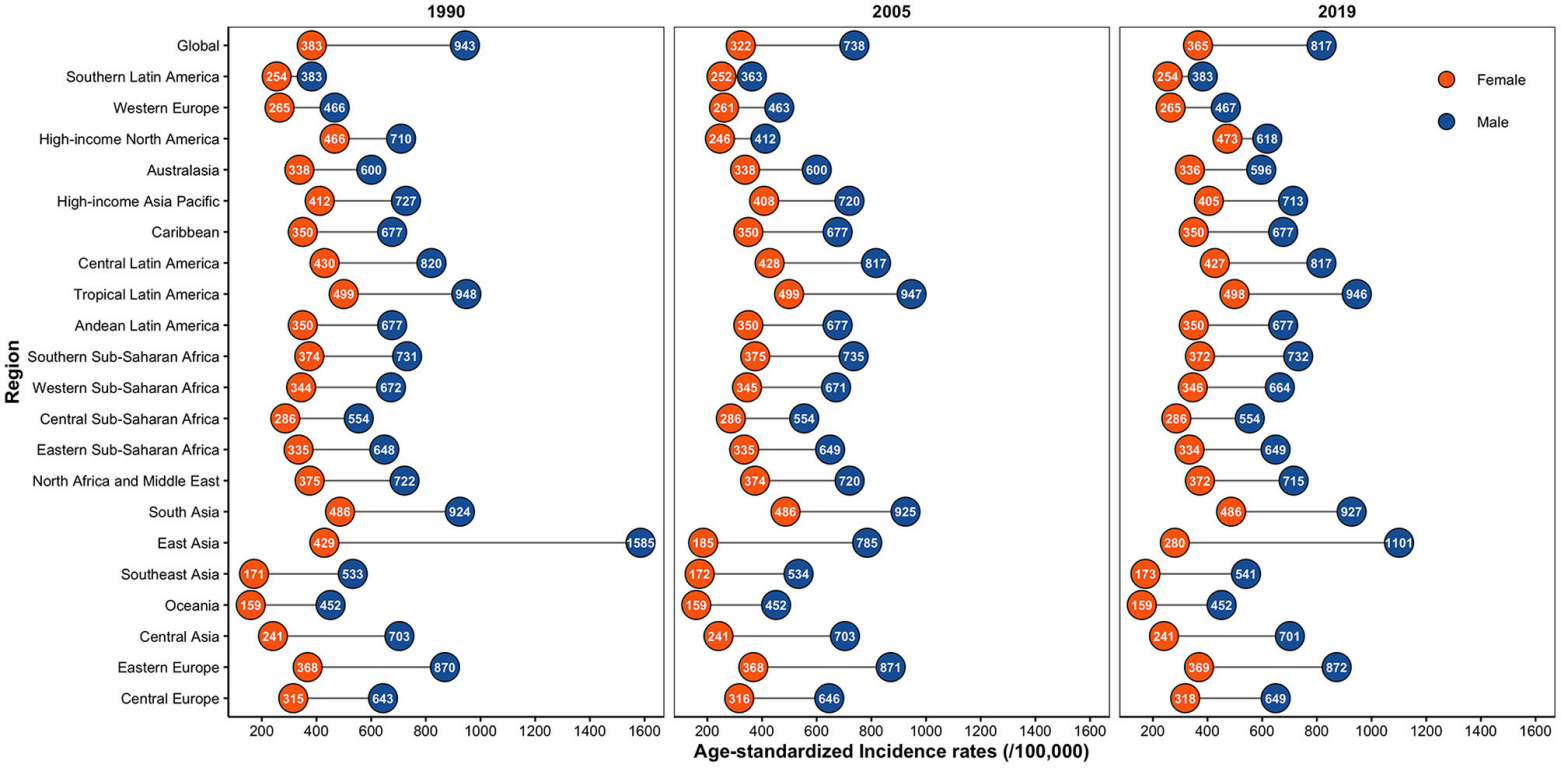
Global sex-specific IOFBs burden by regions in terms of ASIR (per 100,000 persons) in 1990, 2005, and 2019. IOFBs, intraocular foreign bodies; ASIR, age-standardized incidence rate.

In 1990, 2005, and 2019, the ASIR of IOFBs was consistently higher in males than females across all regions (Figure 4). Particularly in East Asia, where the male-to-female ratio reached four times. Notably, among all GBD areas, the low-middle and low SDI regions had the highest ASIR of IOFBs in females, which remained stable from 1990 to 2019 (Figure 3).

## Discussion

Our research reports ASIR and incident cases of IOFBs from 1990 to 2019 in 204 countries and their global distribution by region, country, socio-economic level, age, and sex, as reported in GBD 2019. Although the ASIR of IOFBs decreased with an EAPC of −0.93 globally from 1990 to 2019, it showed an increasing trend after 2008. Furthermore, the incidence of IOFBs varied across regions and countries and was intricately linked to socio-economic development. Notably, there was a significant increase in incident cases of IOFBs in older adults compared to other age groups. Sex-wise, males accounted for the vast majority of IOFBs incident cases. Overall, the global incidence caused by IOFBs cannot be ignored. The economic structure, social structure, and government policy responses of each area primarily determined the trend and outcome of IOFBs in a particular region.

Despite the fact that the ASIR of IOFBs in East Asia remained unquestionably high in 2019, this region has led the world in decreasing the ASIR and incident cases of IOFBs over the last 30 years. China, with the vast majority of land and population, is a deciding factor in this outcome. China's policymaking and economic restructuring are instructive. Initially, in 2009 the Chinese government promulgated “The Interim Provisions on the Supervision and Regulation of Workplace Occupational Health” to ensure prevention, control, and elimination of occupational hazards at a legal level and to clarify the obligation of employers to protect occupational safety of employees, such as providing comprehensive protective equipment (16). Personal protective eyewear (PPE) is by far one of the most cost-effective preventive interventions, according to several studies (8, 17–19). In fact, by using PPE, over 90% of work-related injuries can be effectively prevented (20). However, merely 0.77–39% of IOFBs patients reported wearing safety goggles at the time of the injury (8, 17–19). Another reason for the decrease in IOFBs in China could be related to the ban on fireworks. According to a previous study, fireworks at festivals lead to significant eye damage every year, limiting their use and exposure can lessen eye injuries up to 87% (12). Since 2006, most Chinese cities have implemented fireworks safety guidelines and have enforced bans for environmental protection and personal safety (21). Furthermore, as the Chinese economy has expanded rapidly, agricultural and industrial production methods have shifted from manual labor and traditional manual assembly lines to mechanical automation. The total power of agricultural machinery has increased from 526 million kilowatts in 2000 to more than 1 billion kilowatts in 2019 (22, 23). From 2005 to 2016, China's operational stock of industrial robots steadily increased at an annual rate of 38%, making China the world's largest user of industrial robots (24). Farmers and workers are currently working in safer environments owing to agricultural machinery and industrial robots. While China's IOFBs load remains high, the downward trend offers hope.

SSA, the main distribution area of low and low-middle SDI regions, exhibited relatively low ASIR of IOFBs in 2019, surprisingly showing a high increase in the number of incident cases over a three-decade period. This region also reflected the overall situation in low and low-middle SDI regions, with low social, economic, and educational development. The majority of SSA countries are highly underdeveloped, with large populations living in tribal societies (25). Its economy is primarily agricultural, and more than 60% of the population is comprised of smallholder farmers (26). Over the last two or three decades, an incomplete network of primary industries has been established, absorbing a certain number of farmers (27, 28). This workforce sector participating in primary industrial production, such as mining, has resulted in a rise in trauma cases, including IOFBs. Another factor contributing to the high number of injuries is the illiteracy of grassroots workers, who lack adequate skill training in occupational hazards. As a result, it is critical to provide primary vocational education and appropriate protective gear to manual workers who lack protection awareness. For a long time, political, economic, and ethnic divisions in this region have resulted in conflict. So, war is another major factor worth mentioning (29). War has caused extensive trauma and significantly hampered socio-economic progress, weakened government regulation of employers, and increased the risk of labor injuries. Furthermore, we observed that ASIR of IOFBs continued to rise in high, high-middle, and middle SDI regions after 2008, attributed to regional economic development and workforce changes. Following 2008 global financial crisis, virtual economies such as the financial sector of these countries were severely impacted, while the real economy regained importance and the workforce increased (29). Further investigation is required to determine the precise cause.

In each region, males had a higher ASIR of IOFBs than females, consistent with previous findings (14, 30). Among adults, males and females work in diverse occupations and industries. Many workplaces that are vulnerable to IOFBs, such as construction, welding, carpentry, and mining, are in predominantly male-dominated industries. Notably, IOFBs in children, particularly in boys, are not uncommon (31). Boys have been more active than girls, and they are more likely to be exposed to some hazardous situations, such as fireworks and gunshot toys. As a result, regulations should be strengthened to prevent consumers from using and purchasing hazardous toys. Furthermore, among all GBD regions, low and low-middle SDI regions had the highest proportion of ASIR in females, which remained stable between 1990 and 2019. This phenomenon could be attributed to females accounting for at least half of the labor force in SSA, as well as the country's slow social development (32).

According to our findings, the incident cases of IOFBs in older adults increased from 1990 to 2019. With the population aging, delays in retirement age kept many elders in the workforce. Most of the eye injuries in elders are caused by occupational hazards, especially in developing countries. According to Onakpoya's study in Nigeria, more than 75% of the elderly were still working, and eye trauma in the elderly was most common at work (43.4%) (33). Furthermore, older adults have a higher incidence of visual impairment and blindness due to the presence of age-related eye diseases such as cataracts, glaucoma, and age-related macular degeneration. Poor vision makes them lack the awareness and ability to protect themselves in dangerous situations. In response to the increased incident cases of IOFBs among older adults, public health personnel need to advocate for vision improvement, including treating eye diseases such as cataracts. The social security system should be improved so that older adults have the basic financial security they need to reduce occupational hazards in high-risk jobs.

Our findings have several key implications for health policy makers and researchers. First, the global incidence of IOFBs has been increasing since 2008 and requires urgent attention. Second, there is wide variation in the incidence of IOFBs according to SDI and geographic distribution. Further analysis of these disparities and the development of policies and practices aimed at reducing them are essential. Third, more robust and reliable prevention measures should be evaluated and implemented in regions with the highest incidence of IOFBs (e.g., South Asia, East Asia) and in populations most affected by IOFBs (e.g., the young population aged 15–49 years and males). Fourth, in addition to continued public health efforts targeting regions and populations with high ASIR of IOFBs, there could be important opportunities for IOFBs prevention in regions and populations with significantly increased incident cases (e.g., SSA and older adults).

## Limitations

There were several limitations to this study. As with other analyses in GBD research, data availability influences the uncertainty of IOFBs incidence rates. In areas with missing or sparse data, particularly in developing countries, the modeling framework relies more on covariates and accessibility of data, resulting in greater ambiguity in point estimates. Secondly, some individuals with IOFBs, particularly those with mild clinical symptoms, may not seek medical attention after injury and thus are not represented in the analysis, potentially leading to underestimating the global IOFBs incidence. Lastly, there is a considerable time lag between data collection and database inclusion, resulting in a time lag in evaluating IOFBs.

## Conclusions

Overall, there is a significant upward trend in ASIR and incident cases of IOFBs globally between 2008 and 2019, which makes IOFBs remain a global health challenge. The incidence of IOFBs was strongly related to SDI and varied geographically. Males had a higher ASIR of IOFBs than females. As the population ages, the incident cases of IOFBs in the elderly will further increase in the future, which should be taken into account by policy makers. Our findings can assist governments and healthcare planners in developing practical and targeted policy responses based upon the characteristics of their respective regions. They can also encourage the international community to focus on regions and populations with a higher incidence of IOFBs to reduce the growing global incidence of IOFBs.

## Data Availability Statement

The original contributions presented in the study are included in the article/Supplementary Material, further inquiries can be directed to the corresponding author.

## Author Contributions

QL made contributions to the conception and design of the work, provided administrative support, and analyzed the data in our study. MY collected and organized the data, made contributions to the acquisition, analysis, and interpretation of data, and was a major contributor in writing the manuscript. Both authors read and approved the final manuscript.

## Funding

This study was supported by grants from the Young Talent Program of Gusu Health Project (Grant No. GSWS2020014).

## Conflict of Interest

The authors declare that the research was conducted in the absence of any commercial or financial relationships that could be construed as a potential conflict of interest.

## Publisher's Note

All claims expressed in this article are solely those of the authors and do not necessarily represent those of their affiliated organizations, or those of the publisher, the editors and the reviewers. Any product that may be evaluated in this article, or claim that may be made by its manufacturer, is not guaranteed or endorsed by the publisher.
