# Supplementary material for: Trends and Disparities in the Incidence of Intraocular Foreign Bodies 1990–2019: A Global Analysis
Source: Front Public Health. 2022 Jun 20;10:858455. doi: 10.3389/fpubh.2022.858455 (PMC9253988; doi:10.3389/fpubh.2022.858455)
Supplement: Supplementary file 1 [file Table_1.DOCX]

**Table S1.** **Estimated number and age-standardized rate (per 100,000 persons) of incidence and corresponding trend for IOFBs in 204 countries from 1990 to 2019**

| Country | Number (95% UI) | | 1990-2019 number of change (%) (95% UI) |  | ASIR (95% UI) | | 1990-2019 EAPC (95% UI) |
| --- | --- | --- | --- | --- | --- | --- | --- |
|  | 1990 | 2019 |  |  | 1990 | 2019 |  |
| Afghanistan | 54264.17 (39145.02,74189.24) | 196360.82 (140300.34,268321.54) | 261.86  (245.76,283.26) |  | 504.77 (371.13,678.12) | 516.93 (379.98,697) | 0.07  (-0.12,0.25) |
| Albania | 14721.9 (10623.93,20210.85) | 11122.14 (8169.46,14904.16) | -24.45  (-30.88,-16.99) |  | 427.68 (315.63,574.2) | 426.84 (313.9,574.22) | 0  (-0.2,0.2) |
| Algeria | 128279.24 (91458.48,174767.64) | 224383.46 (163603.7,303922.79) | 74.92  (51.59,96.98) |  | 514.51 (378.01,693.48) | 515.05 (378.69,694.17) | 0  (-0.18,0.19) |
| American Samoa | 147.03  (106.59,202.18) | 168.71  (121.96,232.55) | 14.75  (8.28,22.89) |  | 309.26 (224.57,425.05) | 305.48 (222.14,419.66) | -0.05  (-0.28,0.19) |
| Andorra | 189.55  (135.63,261.31) | 253.39  (183.84,342.35) | 33.68  (19.29,51.2) |  | 329.3 (242.87,442.67) | 325.61 (240.59,438.53) | -0.04  (-0.27,0.19) |
| Angola | 41994.41 (30786.97,57255.41) | 121165.47 (88849.64,164993.24) | 188.53  (184.27,194.17) |  | 421.82 (313.32,568.12) | 413.9 (307.63,557.58) | -0.07  (-0.27,0.13) |
| Antigua and Barbuda | 312.15  (225.49,422.52) | 467.12  (339.63,633.37) | 49.65  (37.66,63.44) |  | 507.69 (372.85,685) | 510.32 (374.64,687.56) | 0.03  (-0.16,0.21) |
| Argentina | 104296.84 (77001.6,140983.06) | 143204.92 (105288.25,192812.86) | 37.31  (30.12,42.12) |  | 317.36 (232.46,427.7) | 317.41 (232.49,427.92) | 0  (-0.23,0.23) |
| Armenia | 16543.64 (12117.72,22729.73) | 13865.71 (10254.27,18826.42) | -16.19  (-21.94,-9.79) |  | 468.7 (345.91,638.97) | 472.03 (349.21,643.59) | 0.03  (-0.16,0.22) |
| Australia | 74925.23 (54787.7,101219.7) | 102263.19 (75351.53,137330.98) | 36.49  (31.69,43.43) |  | 438.2 (323.3,592.23) | 437.25 (322.97,590.48) | -0.01  (-0.2,0.19) |
| Austria | 24487.88 (17824.21,33277.17) | 26198.51 (19200.17,35064.79) | 6.99  (1.54,14) |  | 323.69 (238.57,436.19) | 324.57 (239.31,437.13) | 0  (-0.23,0.23) |
| Azerbaijan | 35686.73 (25738.01,49077.26) | 51107.32 (37467.58,69807.29) | 43.21  (30.32,56.07) |  | 466.6 (344.26,636.23) | 475.72 (351.76,648.25) | 0.08  (-0.11,0.27) |
| Bahamas | 1375.51  (983.01,1880.63) | 1999.97  (1454,2716.13) | 45.4  (34.25,59.01) |  | 509.3 (373.94,687.36) | 508.9 (373.84,686.98) | -0.01  (-0.19,0.18) |
| Bahrain | 3036.12  (2128.8,4240.73) | 9099.78  (6426.75,12783.61) | 199.72  (152.7,255.34) |  | 541.84 (398.61,730.17) | 543.67 (399.93,731.85) | 0.06  (-0.12,0.24) |
| Bangladesh | 553583.06 (397199.98,755427.46) | 841719.45 (612107.72,1138403.53) | 52.05  (38.07,64.66) |  | 519.22 (381.76,700.84) | 509.6 (374.99,687.98) | -0.08  (-0.26,0.1) |
| Barbados | 1312.33  (949,1781.6) | 1469.28  (1066.85,1977.81) | 11.96  (3.47,23.89) |  | 508.85 (373.43,686.49) | 510.39 (374.75,688.21) | 0.01  (-0.18,0.19) |
| Belarus | 46062.2 (33783.29,61995.79) | 39876.11 (29153.02,53943.01) | -13.43  (-18.7,-7.86) |  | 446.08 (327.28,592.39) | 448.97 (329.23,596.23) | 0.02  (-0.18,0.22) |
| Belgium | 38038.58 (27996.28,51270.22) | 40562.62 (29962.57,54018.3) | 6.64  (2.55,11.56) |  | 396.02 (294.5,537.72) | 395.29 (294.12,536.89) | 0.93 (0.74,1.12) |
| Belize | 935.32  (668.06,1271.57) | 2189.79  (1583.37,2951.14) | 134.12  (115.06,151.77) |  | 516.01 (379.65,696.09) | 511.72 (376.46,690.46) | -0.03  (-0.21,0.15) |
| Benin | 18745.19 (13670.73,25687.91) | 51210.48 (37417.28,69961.86) | 173.19  (165,180.61) |  | 411.76 (306.01,555.19) | 416.05 (309.25,560.13) | 0.03  (-0.17,0.24) |
| Bermuda | 321.05  (231.33,439.34) | 305.95  (219.87,412.91) | -4.7  (-13.06,6.11) |  | 510.61 (374.97,689.14) | 511.58 (376.06,691.08) | 0.01  (-0.18,0.19) |
| Bhutan | 3216.44  (2282.62,4407.33) | 4191.39  (3024.59,5704.88) | 30.31  (15.23,42.39) |  | 522.66 (383.93,702.81) | 520.74 (383,700.81) | 0  (-0.18,0.18) |
| Bolivia (Plurinational State of) | 32237.14 (23253.71,43624.5) | 62483.41  (45695,84099.84) | 93.82  (81.05,103.78) |  | 509.83 (374.68,688.29) | 513.29 (377.28,692.26) | 0.03  (-0.16,0.21) |
| Bosnia and Herzegovina | 20206.25 (14681.11,27302.61) | 13047.52 (9545.64,17385.24) | -35.43  (-40.45,-29.42) |  | 426.17 (313.14,572.77) | 424.71 (312.45,570.66) | 0.01  (-0.19,0.21) |
| Botswana | 5221.66  (3756.66,7162.77) | 10412.71 (7623.89,14244.99) | 99.41  (75.26,120.25) |  | 412.99 (307.07,558.45) | 418.79 (311.28,564.78) | 0.11  (-0.09,0.31) |
| Brazil | 1103577.1 (802207.04,1475512.61) | 1622791.74 (1183360.39,2188144.83) | 47.05  (34.06,60.12) |  | 725.63 (536.92,976.56) | 725.96 (537.11,977.1) | 0  (-0.15,0.16) |
| Brunei Darussalam | 1243.59  (892.42,1706.26) | 2119.94  (1521.73,2918.3) | 70.47  (57.54,84.5) |  | 446.28 (329.4,603.74) | 444.36 (327.89,600.86) | -0.01  (-0.2,0.19) |
| Bulgaria | 35562.7 (26244.51,47406.58) | 26477.4 (19479.36,35335.64) | -25.55  (-30.17,-20.95) |  | 424.14 (312.06,569.81) | 426.71 (313.98,573.3) | 0.02  (-0.18,0.23) |
| Burkina Faso | 36555.81 (26779.33,50028.06) | 90811.07 (66487.14,123648.29) | 148.42  (139.93,155.87) |  | 409.83 (304.69,551.94) | 412.94 (306.63,556.83) | 0.02  (-0.18,0.23) |
| Burundi | 22018.79 (16129.44,29954.01) | 48638.62 (35657.98,66282.9) | 120.9  (117.3,124.72) |  | 414.35 (307.47,559.28) | 420.82 (311.98,566.6) | 0.07  (-0.13,0.27) |
| Cabo Verde | 1346.42  (969.82,1831.62) | 2491.2  (1822.13,3357.32) | 85.02  (60.38,108.86) |  | 406.18 (299.66,548.24) | 422.55 (313.9,570.45) | 0.14  (-0.06,0.35) |
| Cambodia | 26699.96 (19267.44,36336.05) | 48502.99 (35279.68,65973.65) | 81.66  (62.75,96.27) |  | 270.71 (199.51,366.05) | 281.87 (206.8,382.21) | 0.14  (-0.11,0.39) |
| Cameroon | 41191.73 (30218.93,55794.3) | 121913.32 (88792.06,167525.23) | 195.97  (184.91,205.29) |  | 416.11 (308.98,560.72) | 418.8 (311.09,563.89) | 0.02  (-0.18,0.23) |
| Canada | 107546.94 (77977.11,146280.5) | 130772.4 (95733.34,174408.67) | 21.6  (15.44,30.55) |  | 386.82 (283.1,522.19) | 386.75 (283.12,521.58) | 0  (-0.21,0.21) |
| Central African Republic | 11071.29 (8092.99,15091.52) | 21894.21 (16048.21,29950.21) | 97.76  (94.13,101.69) |  | 415.96 (308.82,560.96) | 416.22 (309.09,561.06) | 0  (-0.2,0.21) |
| Chad | 23199.69 (17035.85,31405.16) | 63998.71 (46382.02,87390.06) | 175.86  (170.74,181.69) |  | 412.63 (306.73,556.01) | 417.87 (310.27,561.72) | 0.05  (-0.15,0.25) |
| Chile | 43574.09 (31501.51,58975.45) | 57431.55 (42144.81,77380.72) | 31.8  (22.58,42.46) |  | 317.03 (232.2,427.24) | 318.06 (233.03,429.1) | -0.06  (-0.3,0.18) |
| China | 12916424.19 (6505319.31,22023126.67) | 11253673.62 (5703267.71,19037414.86) | -12.87  (-28.97,10.62) |  | 1054.76 (550.59,1754.68) | 718.89 (377.85,1195.56) | -3.44  (-3.59,-3.29) |
| Colombia | 170117.89 (122231.99,230512.3) | 247809.79 (181237.09,334056.48) | 45.67  (35.46,57.27) |  | 510.35 (375.35,689.25) | 510.74 (374.94,688.74) | 0.01  (-0.18,0.19) |
| Comoros | 1850.41  (1350.08,2519.05) | 3072.83  (2268.23,4178) | 66.06  (52,78.8) |  | 416.98 (310.08,561.13) | 420.28 (311.85,566.66) | 0.03  (-0.17,0.23) |
| Congo | 9806.55  (7119.27,13351.6) | 22454.1 (16595.99,30582.24) | 128.97  (113.09,146.75) |  | 415.65 (308.53,560.85) | 418.96 (311.02,564.26) | 0.03  (-0.17,0.23) |
| Cook Islands | 58.18  (42.02,80) | 52.96  (38.18,71.52) | -8.97  (-16.17,1.18) |  | 310.81 (225.67,426.76) | 298.89 (217.55,409.8) | -0.15  (-0.38,0.09) |
| Costa Rica | 15828.01 (11379.85,21387.01) | 24627.36 (18005.26,33156.48) | 55.59  (42.82,68.79) |  | 512.51 (376.8,691.82) | 508.72 (373.48,686.11) | -0.03  (-0.21,0.15) |
| Côte d'Ivoire | 21841.37 (15870.89,29508.97) | 17154.38 (12480.98,23285.39) | -21.46  (-26.77,-15.74) |  | 425.02 (315.64,572.29) | 425.44 (315.62,572.88) | 0.01  (-0.19,0.2) |
| Croatia | 58056.18 (42174.31,78570.83) | 56690.94 (40851.48,76349.05) | -2.35  (-11.32,9.58) |  | 449.46 (326.47,606.5) | 446.01 (325.44,606.76) | 0.01  (-0.17,0.19) |
| Cuba | 2548.69  (1866.85,3447.45) | 4158.83  (3012.1,5682.41) | 63.18  (46.94,74.42) |  | 514.19 (377.81,693.12) | 515.76 (378.82,694.75) | -0.02  (-0.25,0.21) |
| Cyprus | 41028.63 (30121.24,55685.85) | 39515.81 (28996.57,53041.27) | -3.69  (-10.37,3.22) |  | 324.51 (239.05,437.07) | 323.04 (237.82,435.04) | 0.01  (-0.2,0.21) |
| Czechia | 50068.98 (36676.61,68466.42) | 111728.47 (82178.78,153095.58) | 123.15  (114.56,131.79) |  | 404.85 (297.03,546.77) | 407.03 (299.05,549.21) | 0.01  (-0.19,0.21) |
| Democratic People's Republic of Korea | 49205.33 (35563.77,66579.04) | 67384.25 (47547.97,91794.51) | 36.95  (27.9,45.3) |  | 231.4 (167.53,313.41) | 241.41 (175.04,326.94) | 0.18  (-0.09,0.45) |
| Democratic Republic of the Congo | 152948.2 (111529.71,207423.82) | 363612.97 (265715.04,497610.51) | 137.74  (131.52,143.24) |  | 415.86 (308.28,561.09) | 420.07 (311.58,566.37) | 0.04  (-0.17,0.24) |
| Denmark | 16121.76 (11774.69,21779.85) | 16939.53 (12544.65,22483.67) | 5.07  (1.42,10.23) |  | 324.44 (239.21,437.06) | 324.39 (239.32,437.04) | 0  (-0.23,0.23) |
| Djibouti | 2058.03  (1488.41,2819.38) | 5347.47  (3924.51,7306.38) | 159.83  (137.1,185.18) |  | 430.69 (319.84,580.38) | 428.37 (318.71,574.96) | -0.02  (-0.21,0.18) |
| Dominica | 372.23  (268.56,501.48) | 353.54  (258.72,473.27) | -5.02  (-12.71,4.16) |  | 514.67 (376.92,694.16) | 517.26 (380.56,696.83) | 0.01  (-0.17,0.19) |
| Dominican Republic | 36967.64 (26506.27,50189.1) | 57560.42 (41903.63,77802.98) | 55.7  (44.33,67.25) |  | 508.88 (374.48,687.31) | 514.6 (378.28,694.09) | 0.05  (-0.13,0.23) |
| Ecuador | 51493.65 (37072.61,69926.97) | 92056.38 (66990.54,124167.38) | 78.77  (67.38,89.25) |  | 511.52 (376.22,690.78) | 512.37 (376.35,690.95) | 0  (-0.18,0.19) |
| Egypt | 286781.48 (208357.87,386292.53) | 528796.84 (384378.25,715270.15) | 84.39  (75.8,91.61) |  | 516.64 (379.75,696.01) | 518.94 (381.67,699.19) | 0.01  (-0.17,0.2) |
| El Salvador | 26422.17 (18921.15,36121.13) | 31964.21 (23359.01,42851.3) | 20.97  (11.23,28.69) |  | 506.86 (372.47,683.84) | 501.9 (367.97,675.58) | -0.04  (-0.22,0.15) |
| Equatorial Guinea | 1658.87  (1228.99,2254.22) | 6234.99  (4424.76,8550.65) | 275.86  (246.68,303.83) |  | 409.1 (303.77,550.84) | 424.86 (312.98,572.87) | 0.15  (-0.05,0.35) |
| Eritrea | 12179.55 (8836.34,16616.29) | 28730.46 (20988.16,39463.79) | 135.89  (125.18,144.97) |  | 416.88 (309.08,562.64) | 420.07 (311.53,566.94) | 0.03  (-0.17,0.23) |
| Estonia | 6844.01  (5037.59,9139.07) | 5387.23  (3961.09,7272.95) | -21.29  (-25.11,-17.33) |  | 446.92 (327.68,593.45) | 452.06 (331.89,600.27) | 0.05  (-0.14,0.25) |
| Eswatini | 3162.55  (2285.02,4366.89) | 4884.29  (3553.08,6677.31) | 54.44  (42.16,65.2) |  | 411.13 (305.8,555.64) | 416.29 (309.36,562.23) | 0.04  (-0.16,0.25) |
| Ethiopia | 290899.63 (209825.69,398563.24) | 639250.33 (454327.49,871857.07) | 119.75  (112.39,126.06) |  | 600.3 (440.31,801.8) | 602.8 (441.6,806.45) | 0.01  (-0.16,0.18) |
| Fiji | 2343.74  (1694.02,3237.64) | 2873.95  (2084.33,3956.7) | 22.62  (15.72,31.26) |  | 306.99 (223.08,421.38) | 308.22 (223.69,423.45) | 0.02  (-0.22,0.25) |
| Finland | 15966.53 (11534.38,21882.75) | 15881.67 (11573.62,21464.6) | -0.53  (-3.91,4.22) |  | 326.54 (238.34,448.66) | 327.59 (239.11,450.06) | 0.01  (-0.22,0.24) |
| France | 181443.28 (133710.23,243457.89) | 189309.31 (140717.53,252918.77) | 4.34  (-0.12,10.15) |  | 323.13 (238.42,435.81) | 322.55 (238.06,435.05) | -0.01  (-0.24,0.22) |
| Gabon | 3999.61  (2930.61,5436.19) | 7470.26  (5485.33,10179.07) | 86.77  (76.3,96.8) |  | 420.27 (311.84,567.74) | 415.17 (308.22,558.86) | -0.05  (-0.25,0.16) |
| Gambia | 3987.87  (2911.31,5437.68) | 9337.44  (6779.66,12834.34) | 134.15  (127.03,140.06) |  | 421.86 (312.43,567.58) | 417.41 (309.96,561.65) | -0.05  (-0.25,0.15) |
| Georgia | 25601.21 (18926.2,34906.05) | 14966.35 (11567.02,19499.33) | -41.54  (-46.52,-33.88) |  | 466.35 (343.91,636.15) | 444.97 (343.24,582.75) | -0.16  (-0.36,0.03) |
| Germany | 250083.97 (181389.36,339554.33) | 242444.44 (178560.04,321445.84) | -3.05  (-6.91,1.87) |  | 324.4 (239.19,437.01) | 326.05 (240.24,438.61) | 0.01  (-0.22,0.24) |
| Ghana | 60768  (44542.18,82815.33) | 134333.16 (98365.05,184383.99) | 121.06  (109.19,130.56) |  | 417.48 (310.14,562.13) | 414.29 (307.6,558.23) | -0.04  (-0.24,0.17) |
| Greece | 32199.27 (23766.59,42889.27) | 29037  (21467.73,38921.19) | -9.82  (-16.06,-2.91) |  | 322.99 (238.22,435.57) | 322.96 (238.16,435.44) | 0  (-0.23,0.23) |
| Greenland | 242.89  (175.88,335.13) | 217.42  (158.85,291.41) | -10.49  (-16.5,-1.42) |  | 398.22 (291,538.9) | 391.55 (286.44,528.9) | -0.05  (-0.26,0.16) |
| Grenada | 424.55  (306.72,576.59) | 548.54  (399.67,739.94) | 29.21  (16.07,42.59) |  | 511.39 (375.25,689.7) | 518.21 (380.75,697.64) | 0.05  (-0.14,0.23) |
| Guam | 453.54  (328.52,626.43) | 528.51  (382.01,722.08) | 16.53  (8.08,28.26) |  | 316.79 (230.17,435.85) | 311.55 (226.51,427.86) | -0.05  (-0.29,0.18) |
| Guatemala | 39163.33 (28227.73,53342.2) | 93347.87 (66904.31,126403.37) | 138.36  (120.62,152.95) |  | 509.94 (375.12,688.41) | 506.19 (371.39,681.52) | -0.03  (-0.21,0.16) |
| Guinea | 24134.67 (17904.91,32759.08) | 50466.8  (36852.1,68658.79) | 109.11  (103.33,114.02) |  | 413.89 (307.62,557.64) | 412.83 (306.78,556.05) | -0.02  (-0.22,0.19) |
| Guinea-Bissau | 3972.19  (2891.04,5400.28) | 7910.25  (5734.1,10881.95) | 99.14  (91.26,106.07) |  | 412.15 (306.31,555.33) | 414 (307.65,557.48) | 0.01  (-0.19,0.22) |
| Guyana | 4032.55  (2878.15,5475.88) | 4081.37  (2971.72,5497.85) | 1.21  (-4.57,7.9) |  | 511.1 (375.9,690.02) | 511.36 (375.81,689.95) | 0  (-0.18,0.18) |
| Haiti | 31667.87 (22870.78,42779.16) | 65004.94 (47235.87,87464.82) | 105.27  (94.74,113.91) |  | 507.36 (373.45,685.27) | 506.57 (372.1,682.82) | -0.01  (-0.19,0.18) |
| Honduras | 23158.8 (16541.36,31740.69) | 51122.49 (36759.13,68939.03) | 120.75  (103.99,135.07) |  | 510.22 (375.18,688.89) | 506.72 (372.13,682.81) | -0.03  (-0.21,0.15) |
| Hungary | 42772.03 (31637.52,57328.32) | 37481.23 (27250.03,50284.57) | -12.37  (-17.66,-7.19) |  | 423.33 (311.29,569.21) | 424.94 (312.45,571.21) | 0.02  (-0.19,0.22) |
| Iceland | 830.52  (606.74,1119.99) | 1061.43  (785.49,1414.67) | 27.8  (22.47,35.59) |  | 324.4 (239.2,437.19) | 325.42 (239.85,438.16) | 0.01  (-0.22,0.24) |
| India | 6337480.47 (4635570.94,8450718.87) | 10666379.24 (7746067.56,14409067.23) | 68.31  (58.68,76.02) |  | 738.55 (546.35,993.09) | 735.29 (543.99,989.09) | -0.02  (-0.17,0.13) |
| Indonesia | 762691.89 (553389.49,1050720.71) | 1136679.3 (818947.17,1568315.33) | 49.04  (36.89,61.84) |  | 409.6 (298.76,558) | 413.79 (301.16,563.91) | 0.04  (-0.17,0.24) |
| Iran (Islamic Republic of) | 417474.5 (303068.32,566122.32) | 657624.28 (474390.51,894818.66) | 57.52  (32.07,82.13) |  | 733.69 (543.12,986.35) | 733.04 (542.18,986.15) | 0  (-0.16,0.15) |
| Iraq | 87823.84 (63014.53,119567.84) | 228162.66 (163987.85,308587.89) | 159.8  (139.07,178.23) |  | 516.59 (379.86,696.47) | 517.24 (380.27,696.95) | 0  (-0.19,0.18) |
| Ireland | 11516.52 (8468.49,15576.29) | 14931.13 (11058.06,20109.62) | 29.65  (20.38,39.72) |  | 323.45 (238.9,436.23) | 322.34 (238.14,434.9) | -0.01  (-0.24,0.22) |
| Israel | 15956.33 (11716.85,21735.47) | 29044.37 (21664.1,39252.43) | 82.02  (75.93,88.9) |  | 322.01 (237.5,434.41) | 323.5 (238.58,436.05) | 0.02  (-0.21,0.25) |
| Italy | 254094.35 (185204.02,342403.93) | 242580.27 (178590.3,326109.8) | -4.53  (-11.24,3.25) |  | 462.89 (341.82,634.82) | 464.39 (342.84,636.38) | 0.01  (-0.18,0.2) |
| Jamaica | 11994.34 (8607.31,16325.45) | 14894.51 (10831.56,20202.65) | 24.18  (12.69,34) |  | 509.93 (374.96,688.58) | 512.29 (376.55,690.74) | 0.02  (-0.17,0.2) |
| Japan | 785474.62 (578475.3,1053810.15) | 693745.16 (517903.94,921158.65) | -11.68  (-16.22,-5.76) |  | 626.07 (462.86,847.07) | 627.82 (464.23,849.45) | 0.01  (-0.16,0.17) |
| Jordan | 19446.05 (13798.42,26774.04) | 64117.52 (46466.16,86879.72) | 229.72  (197.84,261.75) |  | 521.53 (383.3,701.56) | 526.02 (386.5,707.53) | 0.03  (-0.15,0.21) |
| Kazakhstan | 79385.34 (58139.7,109125.26) | 87357.57 (64685.41,119245.43) | 10.04  (4.88,15.91) |  | 468.95 (345.08,640.08) | 469.25 (345.89,639.9) | 0  (-0.19,0.19) |
| Kenya | 131273.62 (93084.49,180261.97) | 306633.8 (218607.66,413464.27) | 133.58  (120.19,145.67) |  | 600.12 (440.02,802.84) | 600.82 (439.98,804.08) | 0.01  (-0.16,0.18) |
| Kiribati | 220.09  (159.29,301.92) | 360.02  (262.23,496.47) | 63.58  (59.08,68.92) |  | 302.17 (219.83,415.09) | 300.58 (218.64,412.44) | -0.01  (-0.25,0.23) |
| Kuwait | 10447.02 (7375.65,14449.81) | 26619.1 (18689.38,37451.69) | 154.8  (127.74,182.66) |  | 541.77 (398.91,727.67) | 523.92 (385.03,706.01) | -0.12  (-0.3,0.06) |
| Kyrgyzstan | 21271.17 (15410.03,29028.51) | 32045.24 (23500.33,43853.79) | 50.65  (43.51,57.32) |  | 469.21 (345.54,640.17) | 471.27 (347.62,642.83) | 0.01  (-0.18,0.2) |
| Lao People's Democratic Republic | 10965.35 (8014.77,14930.96) | 21193.87 (15366.71,29035.5) | 93.28  (76.11,105.95) |  | 278.89 (205.32,376.3) | 284.71 (209.45,385.3) | 0.07  (-0.17,0.32) |
| Latvia | 11526.61 (8433.09,15419.34) | 7677.24 (5667.68,10300.49) | -33.4  (-36.69,-29.64) |  | 446.14 (327.12,592.45) | 450.01 (330.12,597.43) | 0.03  (-0.16,0.23) |
| Lebanon | 16414.3 (12031.74,22038.71) | 27006.47 (19692.39,36572.76) | 64.53  (47.43,79.03) |  | 511.93 (375.68,688.02) | 511.58 (375.12,688.5) | -0.01  (-0.19,0.18) |
| Lesotho | 7293.89  (5318.55,9976.51) | 9158.65 (6662.25,12511.88) | 25.57  (15.77,33.75) |  | 417.91 (310.71,563.98) | 419.91 (311.83,565.98) | 0.01  (-0.19,0.22) |
| Liberia | 7686.9  (5695.38,10442.71) | 20495.67 (15029.94,28177.69) | 166.63  (152.27,179.88) |  | 416.54 (308.37,560.55) | 421.31 (312.57,567.08) | 0.05  (-0.16,0.25) |
| Libya | 21649.89 (15532.46,29485.68) | 38310.82 (27442.68,52566.02) | 76.96  (49.12,102.69) |  | 526.58 (386.66,708.48) | 518.67 (381.3,698.95) | -0.03  (-0.21,0.16) |
| Lithuania | 16256.74 (11940.4,21848.51) | 11262.88 (8280.81,15055.98) | -30.72  (-34.58,-25.86) |  | 446.41 (327.37,592.94) | 449.43 (329.63,596.91) | 0.03  (-0.17,0.22) |
| Luxembourg | 1216.64  (883.76,1650.3) | 1916.79 (1396.42,2571.68) | 57.55  (52.05,63.6) |  | 324.15 (239.14,437.05) | 324.69 (239.89,437.6) | 0.01  (-0.22,0.24) |
| Madagascar | 47866.91 (34963.98,64951.86) | 112080.36 (82128.13,153304.55) | 134.15  (126.72,140.99) |  | 418.96 (311.3,564.57) | 419.24 (311.61,564.7) | 0  (-0.2,0.2) |
| Malawi | 38153.18 (27895.14,51953.72) | 76082.95 (54828.46,104881.03) | 99.41  (93.42,104.5) |  | 416.5 (309.41,561.15) | 415.63 (308.7,560.28) | -0.01  (-0.22,0.19) |
| Malaysia | 50575.2 (36794.06,69280.61) | 96530.23 (70032.26,132304.36) | 90.86  (75.55,101.47) |  | 284.87 (209.83,384.75) | 290.46 (213.96,393.24) | 0.07  (-0.17,0.31) |
| Maldives | 586.7  (421.18,796.75) | 1876.43 (1300.08,2689.14) | 219.83  (145.33,286) |  | 287.51 (212.45,387.25) | 317.85 (233.95,434.62) | 0.36 (0.11,0.6) |
| Mali | 33826.93 (25014.5,46022.98) | 87197.25 (63384.1,118854.92) | 157.77  (150.41,164.33) |  | 415.26 (308.41,559.58) | 417.82 (310.33,562.09) | 0.02  (-0.18,0.22) |
| Malta | 1110.85  (815.6,1510.71) | 1186.93  (866.57,1600.53) | 6.85  (-0.09,13.08) |  | 300.8 (221.78,408.67) | 302.81 (223.41,411.06) | 0  (-0.24,0.24) |
| Marshall Islands | 131.49  (94.27,180.94) | 180.49  (131.51,250.13) | 37.27  (22.42,49.94) |  | 309.78 (224.61,425.96) | 309.19 (224.79,424.32) | 0  (-0.24,0.23) |
| Mauritania | 8231.74  (6037.39,11171.54) | 16550.6 (12130.89,22609.67) | 101.06  (96,105.38) |  | 417.29 (310.05,561.73) | 416.26 (309.32,560.28) | -0.01  (-0.22,0.19) |
| Mauritius | 3299.67  (2373.21,4536.08) | 3733.91  (2708.53,5074.65) | 13.16  (3.37,26.25) |  | 284.59 (209.08,385.77) | 284.65 (209.39,385.28) | 0  (-0.24,0.25) |
| Mexico | 620163.49 (448250.06,837563.22) | 931987.04 (680656.1,1254822.01) | 50.28  (36.95,64.24) |  | 723.87 (535.85,974.01) | 724.37 (536.38,974.9) | 0  (-0.15,0.15) |
| Micronesia (Federated States of) | 303.76  (218.63,417.1) | 320.52  (230.26,441.94) | 5.52  (-2.75,13.3) |  | 309.44 (224.59,425.51) | 308.42 (224.07,423.51) | 0  (-0.23,0.24) |
| Monaco | 85.99  (62.84,115) | 99.91  (73.85,131.56) | 16.19  (10.15,24.08) |  | 322.53 (237.7,435.18) | 322.42 (238.12,434.51) | -0.01  (-0.24,0.22) |
| Mongolia | 10447.19  (7443.39,14333) | 16689.55 (12297.44,22962.59) | 59.75  (40.03,79.43) |  | 471.94 (348.05,643.18) | 470.79 (347.13,642) | 0  (-0.2,0.19) |
| Montenegro | 2712.31  (1987.9,3655.28) | 2521.53 (1854.08,3365.13) | -7.03  (-12.35,-1.08) |  | 425.76 (312.85,572.23) | 426.07 (313.43,572.83) | 0  (-0.2,0.2) |
| Morocco | 129922.96 (93373.8,176279.59) | 191421.08 (139628.66,260375.59) | 47.33  (35.19,59.89) |  | 511.73 (376.22,690.48) | 513.86 (377.67,692.96) | 0.01  (-0.17,0.2) |
| Mozambique | 51207.14 (37871.43,69262.69) | 117783.01 (85548.88,160915.57) | 130.01  (123.46,136.18) |  | 410.99 (305.15,554.03) | 412.56 (306.51,555.99) | 0.02  (-0.19,0.22) |
| Myanmar | 114340.88 (82795.12,155980.48) | 157001.64 (114970.77,213457.6) | 37.31  (27.69,46.81) |  | 281.95 (207.44,381.39) | 278.19 (204.45,376.26) | -0.05  (-0.3,0.2) |
| Namibia | 5690.12  (4132.37,7761.34) | 10211.43 (7503.12,13890.5) | 79.46  (68,88.78) |  | 417.4 (310.42,563.31) | 416.27 (309.25,561.68) | -0.01  (-0.21,0.2) |
| Nauru | 30.83  (22.59,42.47) | 32.78  (23.7,45.19) | 6.32  (3.31,8.78) |  | 310.27 (225.03,426.52) | 305.17 (221.94,419.18) | -0.06  (-0.29,0.18) |
| Nepal | 97831.19 (70654.73,132355.56) | 157384.61 (113401.35,211718.64) | 60.87  (51.23,68.47) |  | 512.18 (377,691.69) | 502.5 (369.29,676.18) | -0.08  (-0.26,0.11) |
| Netherlands | 39428.32 (28347.22,53177.59) | 40537.38 (29641.66,54064.6) | 2.81  (-2.82,10.34) |  | 264.19 (190.72,358.04) | 263.76 (190.15,357.36) | -0.08  (-0.38,0.22) |
| New Zealand | 21584.91 (15860.13,29137.16) | 26094.67 (19407.25,34740.79) | 20.89  (15.02,29.51) |  | 624.66 (462.33,844.75) | 621.28 (460.37,839.85) | -0.02  (-0.19,0.15) |
| Nicaragua | 19172.88 (13600.05,26395.03) | 34606.78 (25007,46812.99) | 80.5  (61.99,96.39) |  | 507.94 (373.42,685.51) | 510.6 (374.74,688.63) | 0.02  (-0.17,0.2) |
| Niger | 31413.31 (23083.85,43020.88) | 90266.31 (65288.11,123543.03) | 187.35  (179.04,195.62) |  | 418.81 (310.41,563.51) | 416.91 (309.54,562.18) | -0.02  (-0.22,0.18) |
| Nigeria | 522677.34 (380938.79,700068.73) | 1243684.6 (894811.77,1694442.79) | 137.95  (133.1,143.13) |  | 609.91 (447.46,814.28) | 592.16 (434.12,792.59) | -0.11  (-0.28,0.06) |
| Niue | 6.79  (4.96,9.28) | 5.07  (3.66,6.89) | -25.31  (-30.31,-19.36) |  | 307.94 (223.77,423.06) | 307.44 (223.28,422.16) | 0.01  (-0.23,0.24) |
| North Macedonia | 8804.07  (6449.69,11861.49) | 9053.82 (6609.79,12250.62) | 2.84  (-5.69,10.54) |  | 425.79 (313.02,571.9) | 428.27 (315.13,575.23) | 0.02  (-0.18,0.23) |
| Northern Mariana Islands | 157.44  (112.19,221.03) | 135.25  (95.12,185.78) | -14.09  (-26.97,4.17) |  | 317 (230.36,436.55) | 312.15 (226.52,428.41) | -0.02  (-0.25,0.22) |
| Norway | 24385.42 (17481.43,33204.61) | 29093.82 (21028.17,39528.26) | 19.31  (14.75,25.46) |  | 593.44 (427.38,804.38) | 582.24 (421.94,786.09) | 0.19 (0.02,0.36) |
| Oman | 10854.99 (7825.34,14736.71) | 30410.74 (20893.04,43726.74) | 180.15  (135.23,214.85) |  | 550 (404.43,742.17) | 556.79 (409.19,753.75) | 0.08  (-0.1,0.25) |
| Pakistan | 797642.49 (581373.74,1073189.54) | 1654671.73 (1204205.4,2216158.32) | 107.45  (100.51,113.74) |  | 740.27 (548.01,994.83) | 733.59 (543.19,986.1) | -0.03  (-0.19,0.12) |
| Palau | 49.25  (35.5,68.05) | 61.84  (43.98,85.03) | 25.57  (10.69,44.16) |  | 310.02 (225.48,425.83) | 324.6 (236.27,446.13) | 0.14  (-0.09,0.37) |
| Palestine | 10114.37 (7226.35,13877.73) | 26185.62 (18745.43,35692.02) | 158.9  (142.08,175.52) |  | 511.03 (374.58,688.62) | 515.89 (379.28,695.28) | 0.02  (-0.16,0.2) |
| Panama | 12516.97 (9014.26,16878.97) | 21568.24 (15815.91,29120.89) | 72.31  (63.12,82.99) |  | 515.1 (378.95,694.75) | 514.81 (378.41,693.91) | -0.01  (-0.19,0.18) |
| Papua New Guinea | 12321.23 (8881.23,16890.39) | 30540.84 (22298.46,42181.26) | 147.87  (138.73,155.43) |  | 310.79 (225.67,426.8) | 309.31 (224.88,424.45) | -0.01  (-0.24,0.22) |
| Paraguay | 20502.05 (14795.76,27719.82) | 37012.98 (26921.86,50080.44) | 80.53  (67.39,90.98) |  | 514.13 (377.95,693.81) | 515.57 (378.9,695.03) | 0.01  (-0.17,0.19) |
| Peru | 111376.71 (80144.43,151197.1) | 178536.03 (130287.14,242233.26) | 60.3  (47.41,72.51) |  | 511.52 (376.19,690.62) | 513.51 (377.16,691.65) | 0.02  (-0.17,0.2) |
| Philippines | 256048.31 (185082.95,352286.06) | 488995.93 (358535.5,675813.76) | 90.98  (73.01,105.7) |  | 411.97 (299.97,561.22) | 425.67 (313.4,587.88) | 0.14  (-0.06,0.35) |
| Poland | 230851.02 (167100.58,312932.41) | 220434.35 (159651.13,296725.07) | -4.51  (-11.23,1.51) |  | 605.06 (441.94,818.53) | 607.04 (443.37,821.69) | 0.01  (-0.16,0.18) |
| Portugal | 32341.06 (23584.34,43429.85) | 30677.91 (22345.71,40940.84) | -5.14  (-13.47,6.41) |  | 329.16 (240.54,444.52) | 329.85 (239.94,441.79) | 0.01  (-0.22,0.23) |
| Puerto Rico | 18496.55 (13561.32,24886.9) | 16774.53 (12195.81,22388.85) | -9.31  (-15.36,-1.62) |  | 507.78 (372.65,684.29) | 509.33 (373.9,686.74) | 0.01  (-0.17,0.2) |
| Qatar | 2946.41  (2049.04,4181.19) | 21111.41 (14313.45,30695.59) | 616.51  (560.24,655.59) |  | 570.35 (419.07,768.93) | 584.03 (428.59,789.77) | 0.15  (-0.02,0.32) |
| Republic of Korea | 209606.42 (151041.4,284941.51) | 227721.49 (164439.33,305623.65) | 8.64  (-4.36,24.9) |  | 439.02 (324.13,593.49) | 442.81 (326.96,598.61) | 0.03  (-0.17,0.22) |
| Republic of Moldova | 19887.89 (14592.16,26548.25) | 16130.3 (11700.49,22039.49) | -18.89  (-25.54,-13.05) |  | 444.04 (325.82,589.05) | 449.85 (330.09,597.13) | 0.05  (-0.15,0.24) |
| Romania | 97961.59 (72347.9,130260.84) | 74718.26 (54988.16,99993.06) | -23.73  (-29.5,-17.45) |  | 424.09 (311.81,569.87) | 426.09 (313.34,572.59) | 0.02  (-0.18,0.22) |
| Russian Federation | 956415.71 (696949.13,1287556.04) | 883365.78 (650393.08,1201336.76) | -7.64  (-12.36,-2.35) |  | 634.15 (465.2,868.21) | 635.61 (466.5,870) | 0  (-0.16,0.17) |
| Rwanda | 28457.46 (20647.03,38817.24) | 53492.72 (38927.53,73279.14) | 87.97  (78.65,96.35) |  | 414.47 (307.15,559.76) | 414.74 (307.39,560.02) | 0  (-0.2,0.21) |
| Saint Kitts and Nevis | 207.99  (148.63,283.06) | 316.08  (229.37,430.54) | 51.97  (34.38,72.77) |  | 510.84 (375.29,689.63) | 514.03 (378.16,694.02) | 0.02  (-0.16,0.2) |
| Saint Lucia | 693.51  (497.01,946.69) | 923.09  (669.15,1250.79) | 33.1  (16.12,51.85) |  | 508.35 (373.45,686.42) | 513.55 (377.46,692.41) | 0.04  (-0.15,0.22) |
| Saint Vincent and the Grenadines | 561.95  (400.97,765.82) | 588.17  (429.21,790.22) | 4.67  (-7.11,18.71) |  | 513.12 (376.87,691.86) | 516.36 (380,695.57) | 0.02  (-0.16,0.2) |
| Samoa | 490.04  (348.8,675.76) | 645.05  (462.85,890.21) | 31.63  (25.38,39.41) |  | 311.49 (226.21,427.68) | 309.85 (224.78,425.81) | -0.02  (-0.25,0.22) |
| San Marino | 74.55  (54.61,99.97) | 96.06  (71.01,127.73) | 28.85  (22.15,36.99) |  | 324 (239.01,437.06) | 319.45 (235.73,431.46) | -0.05  (-0.28,0.18) |
| Sao Tome and Principe | 473.52  (342.26,644.78) | 884.78  (646.83,1213.52) | 86.85  (71.51,101.17) |  | 416.45 (309.57,560.68) | 420.85 (312.4,567.13) | 0.05  (-0.16,0.25) |
| Saudi Arabia | 87397.52 (63016.23,118151.18) | 224664.17 (157233.62,314782.6) | 157.06  (114.58,193.08) |  | 540.41 (398.18,726.05) | 539.78 (397.12,723.92) | 0  (-0.18,0.17) |
| Senegal | 29882.43 (21774.93,40612.51) | 62890.53 (45970.19,86161.67) | 110.46  (102.03,117.54) |  | 415.31 (308.39,558.54) | 418.33 (310.5,563.61) | 0.03  (-0.18,0.23) |
| Serbia | 39437.38 (29132.44,52927.55) | 34630.78 (25598.49,45990.22) | -12.19  (-15.28,-8.66) |  | 423.43 (311.37,569.12) | 426.96 (313.95,574.05) | 0.03  (-0.17,0.23) |
| Seychelles | 208.74  (150.7,284.27) | 318.63  (230.09,436.87) | 52.64  (35.17,70.87) |  | 286.78 (211.13,387.9) | 296.31 (218.09,402.06) | 0.15  (-0.1,0.39) |
| Sierra Leone | 14596.52 (10801.55,19810.34) | 34690.1 (25331.23,47547.67) | 137.66  (130.44,143.9) |  | 416.04 (308.62,559.9) | 419.1 (311.03,564.04) | 0.05  (-0.16,0.25) |
| Singapore | 14675.79 (10508.49,20158.54) | 25403.46 (18078.55,34807.01) | 73.1  (53.22,95.13) |  | 438.75 (324.17,592.16) | 439.96 (325,594.24) | 0.03  (-0.17,0.22) |
| Slovakia | 22444.45 (16390.1,30288.06) | 22010.37 (16032.44,29750.58) | -1.93  (-10.45,6.12) |  | 423.63 (311.37,569.38) | 425.77 (313.17,572.12) | 0.02  (-0.18,0.22) |
| Slovenia | 8294.22 (6028.29,11153.93) | 7867.22  (5723.15,10534.23) | -5.15  (-11.65,2.67) |  | 418.65 (305.59,561.9) | 422.21 (308.19,566.04) | 0.04  (-0.17,0.24) |
| Solomon Islands | 981.84  (711.42,1346.11) | 1985.41  (1446.98,2744.51) | 102.21  (90.53,113.47) |  | 310.4 (225.59,426.01) | 306.52 (222.98,420.91) | -0.05  (-0.28,0.19) |
| Somalia | 28955.22 (21216.24,39448.91) | 83566.02 (60636.02,114443.74) | 188.6  (180.2,199.93) |  | 420.94 (312.4,566.88) | 420.96 (312.15,568.6) | 0  (-0.2,0.2) |
| South Africa | 221360.23 (159056.12,304272.9) | 349633.17 (252606.63,477496.36) | 57.95  (44.52,69.17) |  | 594.91 (436.59,808.04) | 599.46 (438.98,814.69) | 0.03  (-0.14,0.2) |
| South Sudan | 24344.06 (17667.14,33227.55) | 37510.04 (27517.22,51289.38) | 54.08  (47.64,60.01) |  | 429.94 (319.29,578.11) | 416.97 (310.24,561.48) | -0.1  (-0.3,0.1) |
| Spain | 121910.18 (89347.46,164462.9) | 132664.58 (97602.57,178270.37) | 8.82  (-1.86,20.84) |  | 323.25 (238.3,435.78) | 323.7 (238.85,436.44) | 0.01  (-0.22,0.24) |
| Sri Lanka | 50393.54 (36686.25,69102.56) | 61872.82 (44945.54,84055.11) | 22.78  (15.33,32.3) |  | 284.34 (209.37,384.52) | 280.01 (205.88,378.33) | -0.06  (-0.3,0.19) |
| Sudan | 100315.6 (72358.73,136664.43) | 213628.49 (153337.9,290412.83) | 112.96  (105.09,119.9) |  | 512.28 (377.03,690.67) | 513.66 (378.05,691.21) | 0.01  (-0.18,0.19) |
| Suriname | 2017.86  (1449.49,2737.55) | 2976.07  (2178.66,4005.34) | 47.49  (36.95,60.09) |  | 514.05 (377.61,692.47) | 512.08 (376.17,690.59) | -0.01  (-0.19,0.17) |
| Sweden | 35333.83 (25774.85,48646.66) | 40375.1 (29692.17,55338.07) | 14.27  (11.77,17.56) |  | 437.1 (321.95,604.99) | 437.58 (322.41,605.58) | -0.43  (-0.64,-0.22) |
| Switzerland | 21605.43 (15792.01,29114.71) | 25605.18 (18875.08,33965.76) | 18.51  (13.47,24.9) |  | 320.68 (237.98,430.18) | 320.61 (238.05,430.18) | 0  (-0.23,0.23) |
| Syrian Arab Republic | 64044.91 (45694.51,88131.65) | 74096.96 (53946.15,99647.27) | 15.7  (4.83,29.44) |  | 516.62 (380.03,696.18) | 503.6 (371.16,677.61) | -0.08  (-0.26,0.1) |
| Taiwan (Province of China) | 51013.37 (36443.05,69751.93) | 59529.58 (41613.89,80651.78) | 16.69  (3.94,32.89) |  | 240.85 (174.77,325.81) | 238.55 (173.53,322.72) | -0.04  (-0.31,0.23) |
| Tajikistan | 25460.16 (18305.06,34807.77) | 47640.54 (34789.75,65340.03) | 87.12  (74.9,97.83) |  | 471.05 (347.65,642.27) | 474.52 (350.64,646.51) | 0.03  (-0.16,0.22) |
| Thailand | 168439.52 (121381.14,230520.93) | 203996.91 (146144.5,276102.4) | 21.11  (8.07,37.71) |  | 282.04 (207.43,381.39) | 282.83 (207.96,382.46) | 0.02  (-0.23,0.26) |
| Timor-Leste | 2214.09  (1601.4,3030.98) | 3678.48  (2659.29,5039.9) | 66.14  (58.12,73.52) |  | 288.05 (212,388.95) | 284.31 (209.4,382.79) | -0.03  (-0.27,0.21) |
| Togo | 14493.01 (10502.49,19785.46) | 33146.71 (24326.76,45396.36) | 128.71  (116.52,140.88) |  | 412.95 (306.63,556.29) | 414.62 (308.03,558.52) | 0.01  (-0.19,0.22) |
| Tokelau | 4.68  (3.42,6.32) | 4.21  (3.07,5.75) | -10.05  (-15.12,-4.36) |  | 301.24 (218.61,413.39) | 309.23 (224.75,425.34) | 0.12  (-0.11,0.36) |
| Tonga | 278.47  (200.7,381.59) | 301.63  (219.15,414.69) | 8.32  (2.47,14.3) |  | 302.39 (220.34,414.39) | 302.12 (220.15,414.76) | 0  (-0.24,0.23) |
| Trinidad and Tobago | 6311.68  (4557.04,8515.31) | 7180.1  (5227.88,9688.42) | 13.76  (2.93,25.76) |  | 513.49 (377.4,692.6) | 515.16 (378.71,694.68) | 0.01  (-0.17,0.19) |
| Tunisia | 43360.75 (31201.4,58817.53) | 60777.94 (44201.73,82228.66) | 40.17  (24.08,57.54) |  | 514.31 (378.09,693.31) | 512.45 (376.48,690.77) | -0.02  (-0.2,0.17) |
| Turkey | 311441.5 (224196.05,419945.03) | 441356.18 (318851.02,597519.36) | 41.71  (25.34,56) |  | 515.43 (378.71,694.89) | 516.5 (379.47,695.79) | 0  (-0.18,0.19) |
| Turkmenistan | 17880.88 (12830.97,24362.75) | 25345.22 (18585.02,34710.93) | 41.74  (30.05,53.68) |  | 468.94 (345.71,639.72) | 481.73 (355.67,657.93) | 0.1  (-0.09,0.29) |
| Tuvalu | 27.23  (19.91,37.05) | 37.27  (26.9,51.3) | 36.91  (32.89,40.54) |  | 294.66 (214.88,404.16) | 313.2 (227.49,430.68) | 0.23  (-0.01,0.46) |
| Uganda | 67910.87 (49218.51,92616.39) | 165846.25 (119602.33,227669.49) | 144.21  (139.23,148.13) |  | 417.2 (309.76,561.7) | 414.79 (308.07,558.64) | -0.02  (-0.22,0.19) |
| Ukraine | 324015.71 (238150.37,437386.09) | 264743.21 (192827.59,358876.29) | -18.29  (-23.29,-13.69) |  | 632.22 (463.89,865.09) | 635.44 (466.41,869.83) | 0.02  (-0.15,0.18) |
| United Arab Emirates | 12034.75 (8423.31,16985.52) | 67334.81 (44110.45,99126.33) | 459.5  (353.57,563.19) |  | 565.53 (415.44,760.07) | 566.89 (416.55,761.39) | 0.05  (-0.12,0.22) |
| United Kingdom | 242069.27 (178322.46,329120.2) | 273540.04 (201593.14,369323.34) | 13  (9.8,17.01) |  | 439.1 (325.11,602.12) | 442.03 (327.16,606.06) | 0.03  (-0.17,0.22) |
| United Republic of Tanzania | 101988.25 (74040.94,138916.99) | 230487.79 (169233.82,314780.36) | 125.99  (119.56,132.56) |  | 414.46 (307.74,558.36) | 414.79 (308.13,558.44) | 0  (-0.2,0.21) |
| United States of America | 1565944.6 (1019161.49,2338740.05) | 1763395.61 (1164198.55,2595027.9) | 12.61  (4.26,21.64) |  | 609.87 (399.34,894.74) | 563.18 (367.51,823.53) | 0  (-0.18,0.19) |
| United States Virgin Islands | 549.42  (403.21,744.51) | 488.08 (354.89,649.85) | -11.16  (-16.32,-3.8) |  | 506.86 (372.58,683) | 507.21 (372.21,683.25) | -1.1  (-1.3,-0.91) |
| Uruguay | 9709.89 (7174.88,13060.42) | 10466.12 (7711.22,14001.71) | 7.79  (3.53,11.09) |  | 317.28 (232.37,427.79) | 317.09 (232.23,427.55) | 0  (-0.23,0.23) |
| Uzbekistan | 100559.69 (72329.46,137095.82) | 168716.47 (123537.57,231547.96) | 67.78  (55.33,79.8) |  | 470.16 (346.55,641.25) | 472.34 (348.6,643.82) | 0.02  (-0.17,0.21) |
| Vanuatu | 443.21  (322.07,605.57) | 886.36  (645.19,1218.03) | 99.99  (93.51,106.12) |  | 308.83 (224.38,424.23) | 305.82 (222.47,420.02) | -0.03  (-0.27,0.2) |
| Venezuela (Bolivarian Republic of) | 98151.55 (70622.43,132519.16) | 145564.35 (106606.96,196967.85) | 48.31  (36.02,63.28) |  | 511.78 (376.18,690.78) | 510.27 (375.12,688.66) | 0.01  (-0.18,0.19) |
| Viet Nam | 184912.71 (133186.66,251376.19) | 293051.16 (211625.6,403912.43) | 58.48  (37.1,79.46) |  | 276.86 (203.38,374.49) | 284.71 (209.12,385.38) | 0.1  (-0.15,0.34) |
| Yemen | 66365.04 (47602.81,91817.03) | 164071.72 (118186.9,222291.43) | 147.23  (129.62,162.59) |  | 513.93 (377.91,692.83) | 514.01 (377.88,693.41) | 0  (-0.19,0.18) |
| Zambia | 31513.28 (22699.23,43139.39) | 76168.87 (55397.99,104739.44) | 141.7  (131.12,151.42) |  | 417.16 (309.72,561.73) | 419.05 (310.9,564.84) | 0.02  (-0.18,0.22) |
| Zimbabwe | 41104.8 (29645.96,56535.26) | 62305.96 (45358.33,84753.81) | 51.58  (43.21,59.19) |  | 417.45 (310.69,563.66) | 413.85 (307.53,558.62) | -0.03  (-0.23,0.17) |

IOFBs = intraocular foreign bodies; UI = uncertainty interval; ASR = age-standardized rate; EAPC = estimated annual percentage change.


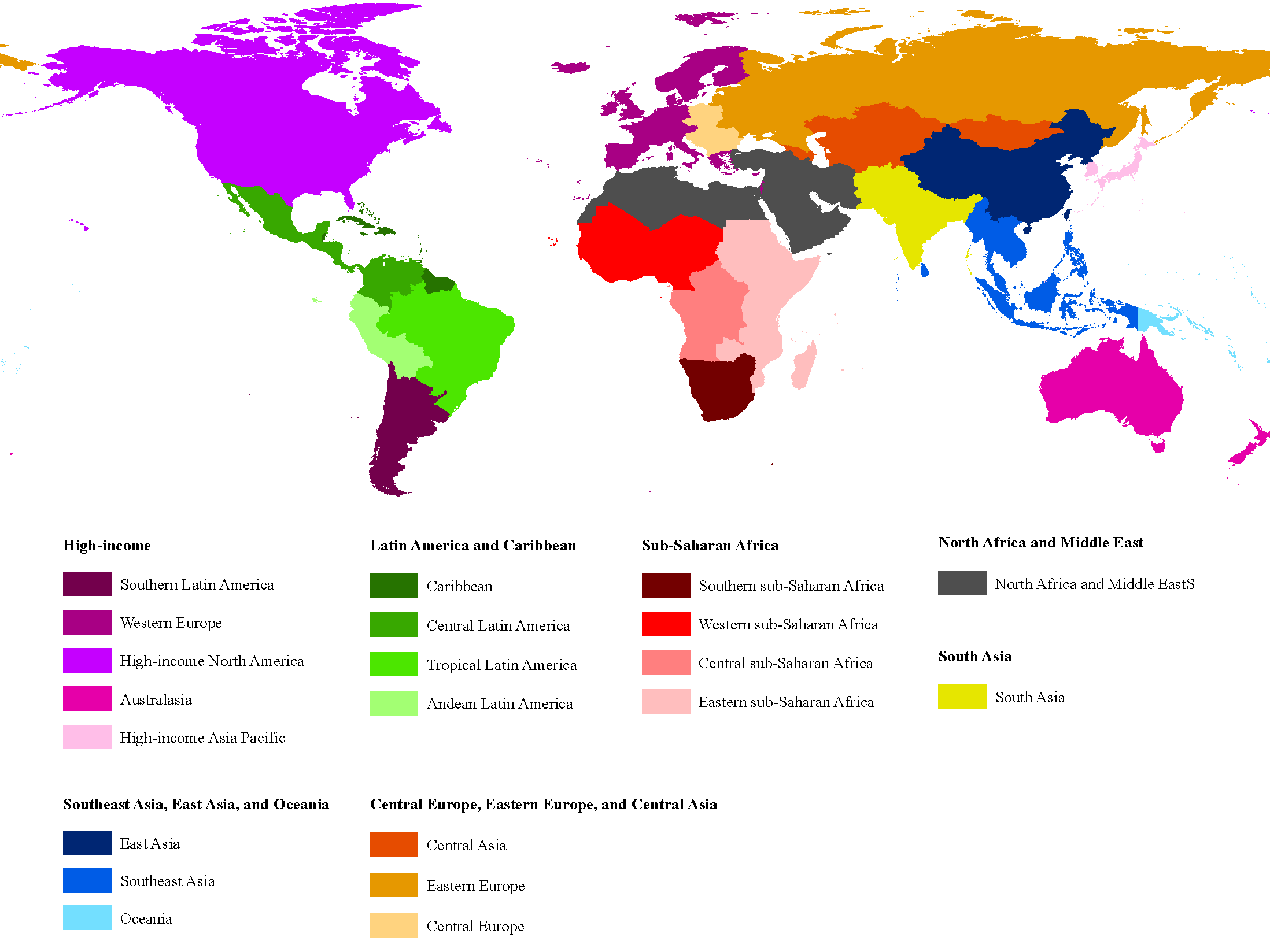


**Figure S1.** **Map of the 21 GBD regions** GBD = Global Burden of Disease Study.


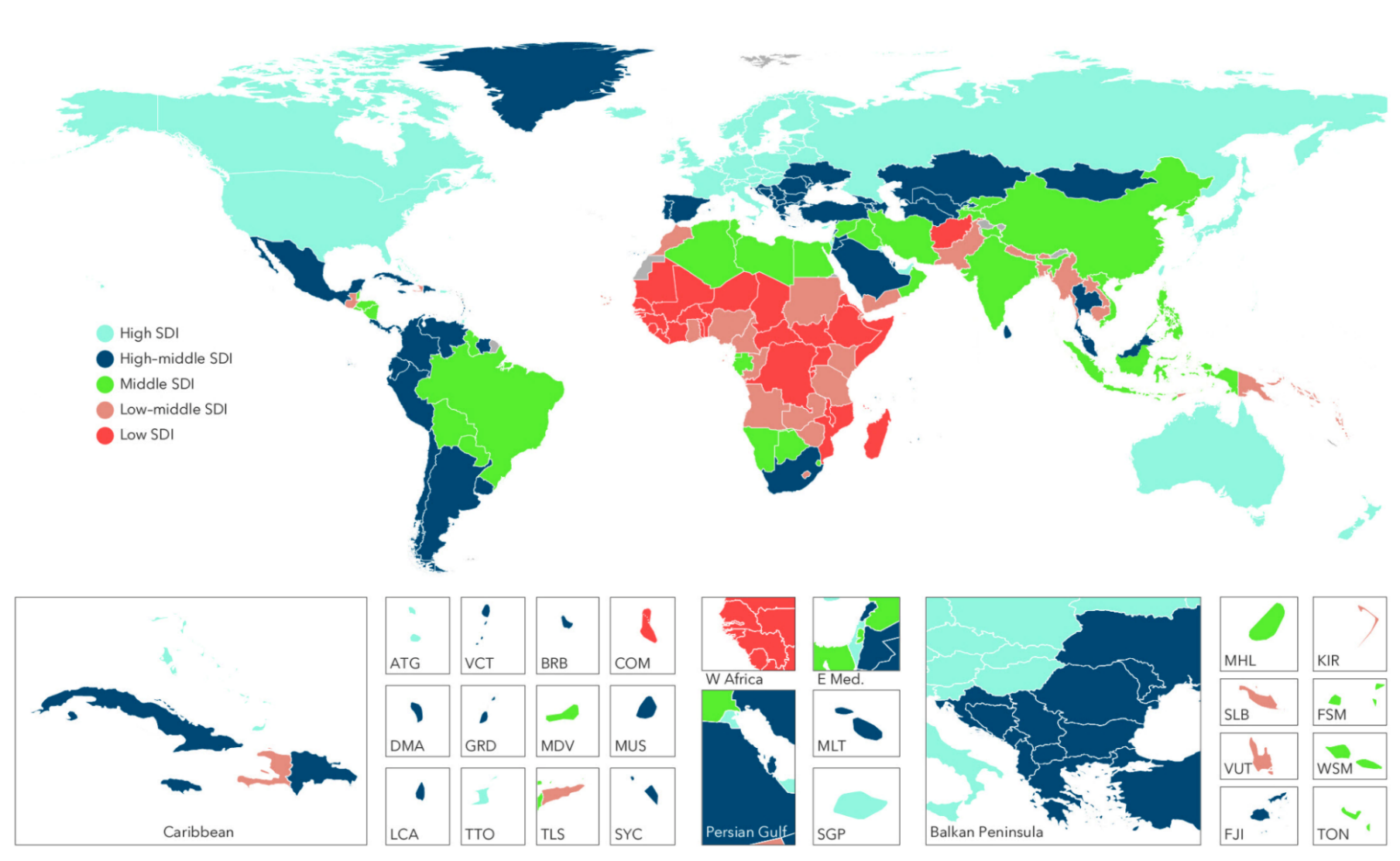


**Figure S2.** **SDI groupings by country** SDI = socio-demographic index.
